# Renal and Cardiovascular Effects of sodium–glucose cotransporter 2 (SGLT2) inhibition in combination with loop Diuretics in diabetic patients with Chronic Heart Failure (RECEDE-CHF): protocol for a randomised controlled double-blind cross-over trial

**DOI:** 10.1136/bmjopen-2017-018097

**Published:** 2017-10-16

**Authors:** Natalie A Mordi, Ify R Mordi, Jagdeep S Singh, Fatima Baig, Anna-Maria Choy, Rory J McCrimmon, Allan D Struthers, Chim C Lang

**Affiliations:** Division of Cardiovascular and Diabetes Medicine, University of Dundee, Dundee, UK

**Keywords:** Sodium-glucose Co-transporter 2 (sglt2) Inhibitors, Heart Failure, Diabetes Mellitus, Natriuresis, Diuresis

## Abstract

**Introduction:**

Type 2 diabetes (T2D) and heart failure (HF) are a frequent combination, where treatment options remain limited. There has been increasing interest around the sodium–glucose cotransporter 2 (SGLT2) inhibitors and their use in patients with HF. Data on the effect of SGLT2 inhibitor use with diuretics are limited. We hypothesise that SGLT2 inhibition may augment the effects of loop diuretics and the benefits of SGLT2 inhibitors may extend beyond those of their metabolic (glycaemic parameters and weight loss) and haemodynamic parameters. The effects of SGLT2 inhibitors as an osmotic diuretic and on natriuresis may underlie the cardiovascular and renal benefits demonstrated in the recent EMPA-REG study.

**Methods and analysis:**

To assess the effect of SGLT2 inhibitors when used in combination with a loop diuretic, the RECEDE-CHF (Renal and Cardiovascular Effects of SGLT2 inhibition in combination with loop Diuretics in diabetic patients with Chronic Heart Failure) trial is a single-centre, randomised, double-blind, placebo-controlled, cross-over trial conducted in a secondary care setting within NHS Tayside, Scotland. 34 eligible participants, aged between 18 and 80 years, with stable T2D and CHF will be recruited. Renal physiological testing will be performed at two points (week 1 and week 6) on each arm to assess the effect of 25 mg empagliflozin, on the primary and secondary outcomes. Participants will be enrolled in the trial for a total period between 14 and 16 weeks. The primary outcome will assess the effect of empagliflozin versus placebo on urine output. The secondary outcomes are to assess the effect of empagliflozin on glomerular filtration rate, cystatin C, urinary sodium excretion, urinary protein/creatinine ratio and urinary albumin/creatinine ratio when compared with placebo.

**Ethics and dissemination:**

Ethics approval was obtained by the East of Scotland Research Ethics Service. Results of the trial will be submitted for publication in a peer-reviewed journal.

**Trial registration number:**

NCT03226457; Pre-results.

**Strengths and limitations of this study**

- Original proof of concept study that aims to explore the effect on diuresis and natriuresis of sodium–glucose cotransporter 2 inhibitors in combination with furosemide.
- Randomised, double-blind cross-over study design.
- Small and single-centre study.
- High participant dropout rate has been factored into power calculations.
- Aims to shed light on the mechanism of the cardiovascular benefits seen in EMPA-REG OUTCOME trial.

## Background

Chronic heart failure (CHF) and type 2 diabetes (T2D) frequently coexist. In population-based studies and in CHF trials, the prevalence of T2D among patients with symptomatic heart failure (HF) is estimated to be between 12% and 41%.^1^ T2D has consistently been shown to be an independent predictor of increased morbidity and mortality in patients with CHF.^2^

For most patients, metformin is the first choice antidiabetic drug in all patients with T2D including those with coincidental HF.^3^ However, metformin alone is often not enough to keep glycaemia under control and there is a frequent need for a second-line antidiabetic drug in patients with HF. Sulfonylureas (SUs) are commonly prescribed in T2D; however, they are associated with weight gain and hypoglycaemia, while there remain concerns that SUs may increase all-cause and cardiovascular (CV) mortality,^4^ although this link is not fully established. Glitazones are contraindicated in New York Heart Association (NYHA) III or IV HF, while their role in milder degrees of HF remains to a certain extent controversial with some observational studies indicating increased hospitalisation or readmission due to HF.^5^ Insulin use has also been associated with increased mortality in patients with CHF.^6^ More recent agents such as the dipeptidyl peptidase-IV (DPP-IV) inhibitors have also, disappointingly, failed to show CV benefit with some concerns raised following the publication of SAVOR-TIMI-53, that they increased HF hospitalisations, although these trials have only been of short duration^7^ and may not apply to all DPP-IV inhibitors. Therefore, it can be concluded that therapeutic options in diabetes mellitus (DM) and HF are limited due to a lack of evidence-based guidelines on the optimal management of such patients. Indeed, international guidelines recognise the evidence gap on the safety and efficacy of drugs used to treat DM in patients with HF as well as the need for agents that will both improve overall glycaemic control and HF outcomes.^8^

### SGLT2 inhibitors and HF

SGLT2 inhibitors are licensed for use in patients with T2D. These oral antidiabetic agents achieve their effects by blocking the low affinity, high capacity type 2 sodium–glucose linked cotransporter (SGLT2), predominantly found in the proximal convoluted tubules of the kidneys, thus causing glycosuria. The recent landmark EMPA-REG outcome study reported a striking 35% relative risk reduction in HF hospitalisations with empagliflozin may provide supportive evidence for beneficial effects of SGLT2 inhibition in the setting of CHF.^9^ A recent analysis of the EMPA-REG study showed that empagliflozin reduced HF hospitalisation and CV death, with a consistent benefit in patients with and without baseline HF.^10^

The mechanisms behind the beneficial effects of empagliflozin on HF and CV death are unknown.^9^ However, given that these effects were seen early in the EMPA-REG trial this would suggest that the effect was not caused simply by altering the atherosclerotic process.^10^

It has been hypothesised that the benefits of SGLT2 inhibitors extend beyond those of the glycaemic parameters of weight loss as promoted by glycosuria, but the effects of SGLT2 inhibitors on non-glycaemia parameters including blood pressure lowering as well as osmotic diuretic and natriuretic effects which may underlie the CV (and renal) benefits.^11 12^

### Renal effects of SGLT2 inhibition and coprescribing with loop diuretics

The renal effects of SGLT2 inhibitors are attracting much recent interest as they may confer renal protection.^13 14^ There are data to support the potential for direct renoprotective actions arising from SGLT2 inhibition including actions to attenuate T1D associated hyperfiltration through an effect on tubuloglomerular (TG) feedback which may have renal protective effects by decreasing glomerular hydrostatic pressure.^13 14^ SGLT2 inhibition has also been shown to attenuate tubular hypertrophy and reduce the tubular toxicity of glucose.^15^ They may also have indirect renoprotective effects through its blood pressure lowering effects and glycaemia lowering effects which could decrease the renal inflammatory and fibrotic response by blocking glucose entry into the cell.^15^ Consequently, there are now several ongoing SGLT2 inhibitor renal outcome trials in T2D, including the CANVAS-R trial (clinicaltrials.gov identifier NCT01989754) and the CREDENCE trial (clinicaltrials.gov identifier NCT02065791). However, neither trial specifically looks at T2DM patients with CHF.

Studies relating to diuresis in the context of SGLT2 inhibitors are surprisingly sparse.^12^ Previous studies with empagliflozin and canagliflozin have demonstrated a 24 hours urinary increase by 300 mL/day after day 1 of treatment but that the daily urinary volume returned to baseline after several weeks.^16 17^ One Japanese case report however, in a non-diabetic patient, described successful treatment of fluid overload that was initially resistant to diuretic therapy, with 5 days of treatment of 50 mg ipragliflozin.^18^

In post hoc analysis of EMPA-REG OUTCOME, Fitchett *et al* reported reduced use of furosemide in patients on the empagliflozin arm, suggesting that these patients reached a relative state of euvolaemia.^10 12^ Heerspink *et al* highlighted that, volume depletion and associated use of loop diuretic is long associated with a prerenal cause of acute kidney injury, and a decrease in loop diuretic may also be relevant in light of the reductions in acute kidney injury, acute renal failure and chronic kidney disease progression endpoints.^12 19^

The renal effects of SGLT2 inhibitors in combination with furosemide in T2D with CHF are not known but given the relative frequency of both comorbidities they are likely to be prescribed concurrently. This underscores the need for a trial to provide detailed acute and long-term information regarding the renal effects of SGLT2 inhibition in combination with loop diuretics, in patients with T2D and stable CHF.

We hypothesise that SGLT2 inhibitors may be able to address the issue of diuretic resistance and may augment the diuretic effects of furosemide in patients with T2D and CHF.

We will recruit patients with diabetic HF taking stable doses of furosemide, or, alternative loop diuretics, with estimated glomerular filtration rate (eGFR) greater than 45 mL/min/1.73 m^2^. This trial will, with careful monitoring, begin the process of uncovering the unrealised potential of this new class of drug which, for the reasons outlined above, is poised to become the second-line antidiabetic agent of choice in patients with HF.

## Methods: participants, interventions and outcomes

### Trial design

**Figure 1:**
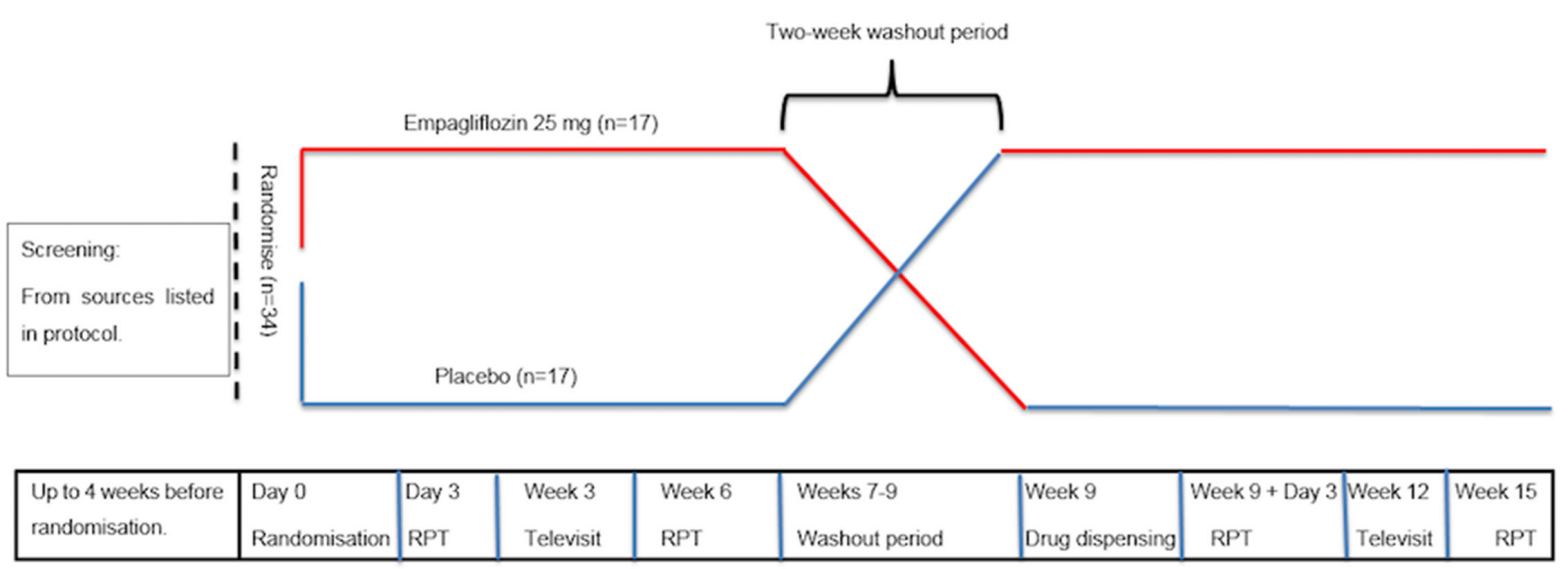
RECEDE-CHF (Renal and Cardiovascular Effects of SGLT2 inhibition in combination with loop Diuretics in diabetic patients with Chronic Heart Failure) trial design.

The RECEDE-CHF (Renal and Cardiovascular Effects of SGLT2 inhibition in combination with loop Diuretics in diabetic patients with Chronic Heart Failure) trial is a single-centre phase IV, randomised, double-blind, placebo-controlled, cross-over trial conducted in NHS Tayside, Scotland, to compare the SGLT2 inhibitor empagliflozin 25 mg with placebo. Participants will be enrolled in the trial for a period of between 14 and 16 weeks. The trial design is summarised in figure 1 and table 1.

**Table 1:** RECEDE-CHF trial protocol

Visit	Visit 1* (screening)	Visit 2* (baseline/randomisation)	Visit 3	Visit 4 (televisit)	Visit 5	Two- week washout period	Visit 6	Visit 7	Visit 8 (televisit)	Visit 9 (final visit§)
Week	Up to 4 weeks pre visit 2	Day 0	Day 3 (±2 days)	Week 3 (±3 days)	Week 6 (±3 days)		Week 9 (±3 days)	Week 9+3 days (±2 days)	Week 12 (±3 days)	Week 15 (±3 days)
Informed consent	X									
Inclusion/exclusion criteria	X	X					X	X		
Medical history	X									
Demographics	X				X		X			X
Vital signs	X	X	X		X		X	X		X
Safety bloods	X	X	X		X		X	X		X
Research bloods		X	X		X		X	X		X
Genetic blood sample†		X								
uPCR/uACR		X	X		X		X	X		X
Urine pregnancy test‡	X	X	X		X		X	X		X
24 urinary collection			X		X			X		X
Renal physiology pest			X		X			X		X
Drug dispensing		X					X			
AE assessment		X	X	X	X		X	X	X	X
Record/review medications	X	X	X	X	X		X	X	X	X
Drug compliance check			X	X	X			X	X	X

*Visits one and two combined into one visit where able.

†Only to be taken if participant consent given.

‡Testing on women of childbearing potential or who do not abstain from sex or use effective contraception.

§If the participant wishes to withdraw prematurely or at the principal investigator’s discretion, all study procedures will be conducted as though the final visit, if participant agrees.

AE, adverse events; RECEDE-CHF, Renal and Cardiovascular Effects of SGLT2 inhibition in combination with loop Diuretics in diabetic patients with Chronic Heart Failure; uACR, urine albumin/creatinine ratio; uPCR, urine protein/creatinine ratio.

At the screening visit, following informed consent, an initial medical history and clinical examination will be performed and concomitant medication will be recorded. Participants will have bloods taken for safety analysis and vital signs will be checked to confirm eligibility prior to enrolment. An assessment of suitability of the trial for the potential participant will be undertaken by the principal investigator (PI) or medically qualified delegate.

Should the participant meet the inclusion criteria and have no exclusion criteria identified, they will return for the baseline/randomisation visit at the Clinical Research Centre (CRC), Ninewells Hospital, Dundee, within 4 weeks postscreening visit. Where able the screening visit and randomisation visits will be combined.

At the randomisation visit participants will undergo safety blood tests, vital signs and study medication will be dispensed (either empagliflozin 25 mg or placebo).

Participants will continue on study medication, empagliflozin 25 mg or placebo, once daily, for a period of 6 weeks. Participants will be educated on the symptoms of hypoglycaemia and given a written action plan on how to manage it in the unlikely event that it occurs.

Participants will return to the CRC 3 days (±2 days) post randomisation, for a study day where they will have safety and research bloods drawn, vital signs recorded and will undergo renal physiological tests (RPTs). Further details of these RPT are described below.

Participants will then return again at week 6 for a study day where they will undergo RPT again, safety and research bloods will be drawn and vital signs recorded. Participants will terminate the study drug, either empagliflozin 25 mg or placebo, at this visit and will return to the CRC at the end of the 2-week washout period (week 9).

At week 9, participants will have safety and research bloods drawn, vital signs recorded, and new study medication dispensed. The RPT will then be repeated at the same intervals, at week 9+3 days (±2 days) and at week 14 for the final study day. Participants will then terminate study drug, either empagliflozin 25 mg or placebo.

### Study population

Thirty-four patients with underlying diabetes and well-controlled CHF will be recruited from a range of sources. The local Tayside database of the systems biology study to tailored treatment in CHF (BIOSTAT) database consisting of around 1800 patients with HF who have previously consented to be approached for future research. The investigators may also recruit from NHS Tayside diabetes and/or HF clinics, and SHARE The Scottish Health Research Register, where participants have preconsented to be invited for research. It is anticipated it will take up to 18 months to recruit the 34 patients for randomisation with approximately 80–100 being consented into the screening trial. We anticipate a screen failure rate of 20% and a dropout rate of 35% after recruitment based on previous HF studies within this research group and the high intensity of the RPT days which will occur on four occasions in total.

### Eligibility

Patients will be eligible if they:

- Aged 18–80 years with previously diagnosed T2DM;
- Are diagnosed with NYHA Functional class II–III HF with prior echocardiographic evidence of left ventricular systolic dysfunction (LVSD);
- On stable doses of furosemide, or alternative loop diuretic for 1 month;
- Stable T2D (HbA1c, in the last 3 months, of 6.5 % ≤ and ≤10.0%);
- Point-of-care B-type natriuretic peptide (BNP) >100 pg/mL;
- eGFR ≥45 mL/min/1.73 m^2^;
- Have stable HF symptoms for at least 3 months prior to consent;
- On stable HF therapy for at least 3 months prior to consent;
- Have not been hospitalised for HF for at least 3 months prior to consent.

Patients will be excluded if they:

- A diagnosis of chronic liver disease and/or liver enzymes that are twice the upper limit of normal;
- Systolic BP of <95 mm Hg at screening visit;
- eGFR <45 mL/min/1.73 m^2^;
- Patients on thiazide diuretics;
- Malignancy (receiving active treatment) or other life threatening disease;
- Pregnant or lactating women;
- Patients with difficulty in micturition, for example, severe prostate enlargement;
- Patients who have participated in any other clinical trial of an investigational medicinal product within 30 days;
- Patients who are unable to give informed consent;
- Any other reason considered by the physician to be inappropriate for inclusion.

### Randomisation and treatment allocation

After successful screening for eligibility and safety, participants will be randomised to either empagliflozin 25 mg/placebo or placebo/empagliflozin 25 mg in a double blind fashion in this cross-over study.

The double-blind medication (empagliflozin or placebo) will be prepared, packaged and labelled by our onsite clinical trials pharmaceutical manufacturer. Randomisation will be carried out by our dedicated clinical trials pharmacy using block randomisation. They will use a validated randomisation programme and will securely backup both the randomisation seed and the randomisation allocation and have it available in the onsite 24 hours emergency unblinding facility.

Participants will be given blinded medication. At the randomisation visit they will be dosed with either empagliflozin 25 mg (6 weeks) or matched placebo (6 weeks) to continue once daily, with a 2 week washout period between each arm. Participants and their bloods (including urea and electrolytes, liver function tests and full blood count) will be monitored as per trial schedule and medication stopped if concerns arise. If the trial drug needs to be stopped due to intolerance or adverse events (AEs), they will remain in the trial in order to do an ‘intention to treat’ analysis.

### Trial outcomes

The primary aim/objectives will be to assess whether empagliflozin (SGLT2 Inhibitor) can augment the diuretic effects of loop diuretics in diabetic patients with mild CHF with LVSD, as measured by urinary volume, compared with placebo.

The secondary aims/objectives are to assess the effect of empagliflozin (SGLT2 inhibitor) on natriuresis when used with loop diuretics in diabetic patients with mild CHF with LVSD as measured by urinary sodium excretion, to measure the safety of add-on SGLT2 inhibitor therapy versus placebo on top of loop diuretics as measured by serum creatinine and eGFR, to assess effects of empagliflozin on protein/creatinine ratio, albumin/creatinine ratio and on the renal biomarker, cystatin C.^20^

### Renal physiology tests

Patients will attend the CRC, Ninewells, Dundee on four separate study days (two while in each arm of the trial). On each study day, patients will present themselves to our CRC, following an overnight fast. Two days before presenting to the CRC, patients will be required to follow a 2 g sodium and 2 L fluid/day controlled diet. A 24 hours urinary collection will be requested the day prior to presentation at the CRC for urinary volume and sodium. Patients will be asked to take their morning usual medications except their investigational medicinal product (empagliflozin or placebo) and furosemide (or equivalent loop diuretic).

An intravenous cannula will be placed in each arm for subsequent infusion and blood sampling. Bloods will be drawn for measurement of plasma N-terminal proBNP and cystatin C using standard protocols. A 15 mL/kg oral water load will be administered over a 15 min period. Thereafter, at 30 min intervals, patients will be requested to void urine until the end of the study period. The volume of urine passed will be measured and an aliquot stored for later analysis. On each occasion, the volume of urine passed will be measured, and an equal volume of water given to drink. In this way, a steady state diuresis will be established over approximately over 3 hours, avoiding the need for catheterisation. The last 30 min urinary collection during this stabilisation period will be taken as baseline. At +150 min, each patient will receive an oral tablet of either empagliflozin 25 mg or placebo. At +210 min, patients will be given a bolus of intravenous furosemide at half their total daily dose.

**Figure 2:**
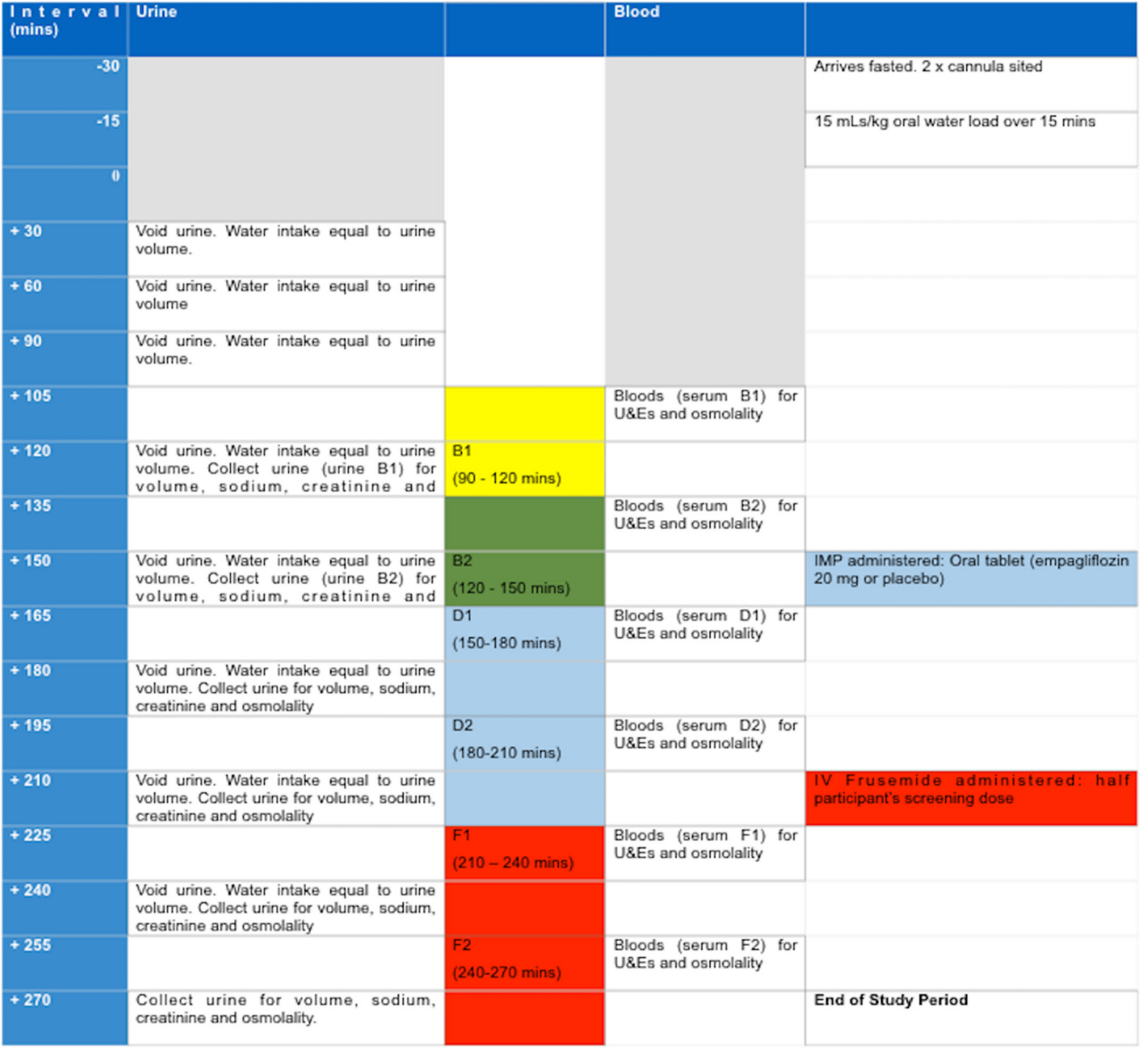
Protocol for renal physiology test days.

Heart rate and blood pressure will be displayed continuously on an ECG oscilloscope and the blood pressure measured every 60 min. Venous blood will be obtained at the midpoint of each clearance period for measurement of serum sodium, osmolality and creatinine. Figure 2 outlines the protocol for the RPT test day.

Intravenous furosemide will be administered to eliminate variable furosemide gut absorption and for practical reasons to complete the study within the set time frame due to the reduced time for the peak onset of diuretic effect with intravenous administration.

Data on the effect of SLGT2 inhibitors and the participant’s usual oral diuretic regime will be gained by the collection of 24 urinary samples on the days before the RPT.^21^

### Tolerability

We will monitor for tolerability of the study medication. One of the inevitable side effects of empagliflozin and other SGLT2 inhibitors with the promotion of glycosuria is urinary tract infections. This typically presents itself as genital mycotic infection, typically *Candida albicans*, which is usually straightforward to treat. However, if patients were to present with infection unresponsive to standard treatment then the trial drug would be discontinued. They will also be warned of the side effects including hypoglycaemia with a written action plan on how to recognise and treat in the unlikely event of a hypoglycaemic episode.

### Sample size and power calculations

Power calculations were performed by the University of Dundee’s Senior Medical Statistician and are based on our previous data in patients with CHF^21^ as well as more contemporary data.^22^ The sample size is based on the mean furosemide-induced urinary volume and sodium excretion of 920 mL/hour (SD=250) and 300 µmol/min (SD=60) respectively. A 20% increase is expected in these parameters following SGLT2 inhibition. This increase in urinary volume is based on published percentage increases in urinary volume in patients with T2D that range from 11% to 33% depending on the dose of the SGLT2 inhibitor as shown by List and colleagues^23^ in the available data with dapagliflozin (dapagliflozin 2.5 mg: 11% increase in 24 hours urine volume; dapagliflozin 5 mg: 22%–11% increase in 24 hours urine volume; dapagliflozin 10 mg: 24%–11% increase in 24 hours urine volume; dapagliflozin 20 mg: 27%–11% increase in 24 hours urine volume and dapagliflozin 50 mg: 33%–11% increase in 24 hours urine volume). There is no data of SGLT inhibition together with loop diuretics. As described previously the Japanese case report of a patient with diuretic-resistant HF in whom fluid overload was successfully treated with the SGLT2 inhibitor, ipragliflozin, where a striking 50% increase in urinary volume (following treatment with 50 mg daily of oral ipragliflozin for 5 days) was described.^18^ Based on the above, it was determined that a 20% increase in furosemide induced increase in urinary volume and sodium excretion will be reasonable.

With an alpha 0.05 and power of 90%, 22 participants per arm are required, assuming 35% dropout. Since this trial is using an AB/BA cross-over design, a total of 34 participants will be required, as each participant will be exposed to both arms of the trial. The rationale for the high dropout rate is due to the high intensity RPTs that will occur on four occasions for the participants, from 09:00 to 14:00 hours we feel that anything less than a 35% dropout rate would be conservative.

## Methods: data collection, management and analysis

### Data collection

The data will be collected by the PI or delegate on a paper case report form (CRF) with subsequent transcription to an electronic CRF. Electronic storage will be in an encrypted form on a password protected device. The medical notes will act as source data for medical history and blood results. Any data relating to general medical history will be documented in the notes.

### Statistical plan

Participants will be allocated to treatment groups at random. Descriptive statistics will be calculated for all data and reported as mean (SD) for continuous data and n (%) for categorical data. For statistical evaluation of repeated measurements, analysis of variance will be used. p<0.05 will be taken as the level of statistical significance. The study is powered based on the primary outcome. In order to examine differences in patient characteristics between study groups, between-group comparisons will be assessed using independent t-tests or analysis of variance (ANOVA). Within-group comparisons will be conducted using paired sample t-tests or repeated-measures ANOVA if data meet parametric assumptions.

## Methods: monitoring

### Data monitoring

A data monitoring committee is not considered necessary as this is a relatively small trial. Close supervision of the PI/clinical research fellow will be conducted by an experienced chief investigator supported by a senior trial manager from the Tayside Clinical Trials Unit.

The purposes of trial monitoring are to verify that the rights and well-being of human subjects are protected, the reported trial data are accurate, complete and verifiable from source documents and the conduct of the trial is in compliance with the currently approved protocol/amendment(s), with good clinical practice and with the applicable regulatory requirement(s). The Sponsor will determine the appropriate extent and nature of monitoring for the trial and will appoint appropriately qualified and trained monitors. The monitor will communicate any monitoring findings to both the CI and PI and the Sponsor.

### Identifying, recording and reporting AEs

Participants should be instructed to contact a member of the trial team at any time after consenting to join the trial if any of the above symptoms develop. All reported AEs that occur after joining the trial will be recorded in detail in the CRF AE log. In the case of an AE, the Investigator should initiate the appropriate treatment according to their medical judgement. Participants with AEs present at the last visit must be followed up until resolution of the event.

The CI or delegate will ask about the occurrence of AEs and hospitalisations at every visit during the trial. AEs will be recorded on the AE Log in the CRF. Serious adverse events (SAEs) will be submitted on an SAE form to the TASC Pharmacovigilance Section within 24 hours of becoming aware of the SAE. SAEs will be initially assessed for causality and expectedness by the Investigator. The Sponsor will make the definitive assessment on expectedness. The evaluation of expectedness will be made based on the knowledge of the reaction and the relevant product information (summary of product characteristics).

### Confidentiality

All laboratory specimens, evaluation forms, reports and other records will be identified in a manner designed to maintain participant confidentiality. All records will be kept in a secure storage area with limited access to trial staff only. Clinical information will not be released without the written permission of the participant, except as necessary for monitoring and auditing by the Sponsor, its designee or Regulatory Authorities. The CI and trial staff will not disclose or use for any purpose other than performance of the trial, any data, record or other unpublished, confidential information disclosed to those individuals for the purpose of the trial. Prior written agreement from the Sponsor or its designee will be obtained for the disclosure of any said confidential information to other parties.

### Ethics and dissemination

Ethics approval was obtained by the East of Scotland Research Ethics Service. This trial has been funded by the British Heart Foundation who have peer reviewed the grant application. Additional peer review of the protocol occurs via the Sponsorship Committee.

The clinical trial report will be used for publication and presentation at scientific meetings. Trial investigators have the right to publish orally or in writing the results of the trial.

Prospective registration will be obtained via clinicaltrials.gov before the enrolment of patients.

Should any protocol modifications arise, these will be decided by the CI on discussion with the PI, which would then be escalated to the trial sponsor, who will decide if further notification to the relevant authorities is required.

### Access to data

Ownership of the data arising from this trial resides with the trial team and their respective employers. On completion of the trial, the trial data will be analysed and tabulated, and a clinical trial report will be prepared.

## Discussion

SGLT2 inhibition is an innovative strategy for the management of T2D, where historically, antihyperglycemic interventions have focused on restoring B-cell activity, insulin sensitivity, or tissue glucose uptake to normalise plasma glucose levels.^12^

In this proof of concept trial, we hypothesise that the benefits of SGLT2 inhibitors extend beyond those of their metabolic (glycaemic parameters and weight loss) and haemodynamic parameters; that the effects of SGLT2 inhibitors as an osmotic diuretic and on natriuresis may underlie the CV and renal benefits.

While the encouraging renal and CV outcomes demonstrated in EMPA-REG might be explained by the modest but cumulative effect of blood pressure lowering (mean reduction in systolic BP ~3 mm Hg), HbA1c reduction (0.3%), and weight reduction (~1 kg), it is difficult to ignore the possibility that an additional mechanism may also be at work.^19 24^ For example, Mudaliar *et al* hypothesise that empagliflozin may also improve renal fuel energetics and efficiency, providing more energy efficient oxygen consumption and thereby potentially less hypoxic stress on the diabetic heart and kidney.^24^

We have also described that SGLT2 inhibitors may augment the effect of loop diuretics. It is noteworthy that osmotic diuretics such as mannitol have been used alone or in combination with loop diuretics such as furosemide to promote diuresis in patients undergoing intracranial surgery^25^ and in the postoperative period to prevent acute kidney injury.^26^ In CHF, mannitol was reported to promote effective diuresis in a single-centre study in the USA.^27^ Importantly, in all these settings, mannitol, which is a potent osmotic diuretic when used in combination with furosemide, was shown to be safe and did not result in renal failure or electrolyte disturbances.

By detailing urinary volumes and sodium excretion via RPTs at two points 3 days and 6 weeks into the investigational medicinal product, the trial’s primary aims to assess the change to these markers representative of diuresis in patients on empagliflozin and when compared with placebo. The effects on natriuresis, proteinuria, albuminuria, cystatin C will also be studied as will the safety of add-on SGLT2 inhibition versus placebo.

### Limitations

While power calculations have been conducted to calculate the sample size, an obvious limitation is that this is a small single-centre trial. The proposed trial will require participants on four occasions to undergo detailed renal physiological tests, (from 09:00 to 14:00 hours) as such a dropout rate has been factored in of 35%.

HF is a dynamic disease; its natural history is one of the flux as the patient’s intravascular volume changes, their loop diuretic requirement may also fluctuate. However, as stipulated on the inclusion criteria, we will be taking patients with HF with evidence of previous LVSD, but with stable symptoms and medications for 3 months, who have not had a hospital admission for HF within this same time frame.

## Conclusion

While the osmotic diuretic hypothesis^11^ is frequently discussed in relation to the renal and CV outcomes with SGLT2 inhibition, literature on the effect of SGLT2 inhibitors on diuresis is currently limited. At time of writing, no studies have been performed to assess the effect of loop diuretics when used in combination with SGLT2 inhibitors. This proof of concept trial will aim to shed light on the mechanism of the CV and renal outcomes demonstrated in the recent EMPA-REG study by documenting the influence of the SGLT2 inhibitors when used in combination with a loop diuretic on urinary volumes and natriuresis when compared with placebo.

Further intent of this proposed study is to obtain data that might be relevant to the design of future studies in which SGLT2 inhibitors may be considered as an adjunctive agent to loop diuretics in patients who experience diuretic resistance. Although data are currently limited, since HF and T2D are frequent comorbidities, it is probable that physicians may find patients requiring both SGLT2 inhibitor therapy and furosemide concurrently. Only by studying this, can these fundamental issues be addressed and, perhaps, inspire a change in practice.

## Supplementary Material

Reviewer comments

Author's manuscript
