# Supplementary material for: Renal and Cardiovascular Effects of sodium–glucose cotransporter 2 (SGLT2) inhibition in combination with loop Diuretics in diabetic patients with Chronic Heart Failure (RECEDE-CHF): protocol for a randomised controlled double-blind cross-over trial
Source: BMJ Open. 2017 Oct 16;7(10):e018097. doi: 10.1136/bmjopen-2017-018097 (PMC5652490; doi:10.1136/bmjopen-2017-018097)
Supplement: Author's manuscript [file bmjopen-2017-018097.draft_revisions.pdf]

BMJ Open is committed to open peer review. As part of this commitment we make the peer review history of every article we publish publicly available.

When an article is published we post the peer reviewers' comments and the authors' responses online. We also post the versions of the paper that were used during peer review. These are the versions that the peer review comments apply to.

The versions of the paper that follow are the versions that were submitted during the peer review process. They are not the versions of record or the final published versions. They should not be cited or distributed as the published version of this manuscript.

BMJ Open is an open access journal and the full, final, typeset and author-corrected version of record of the manuscript is available on our site with no access controls, subscription charges or pay-per-view fees (http://bmjopen.bmj.com).

If you have any questions on BMJ Open's open peer review process please email editorial.bmjopen@bmj.com

BMJ Open

Research into the Renal and Cardiovascular Effect of Sodium-Glucose co-Transporter 2 (SGLT2) inhibition in combination with Loop Diuretics in Diabetic patients with Chronic Heart Failure (RECEDE-CHF): A Randomised Controlled Double-Blind Crossover Trial: Trial Rationale and Protocol

Journal:	*BMJ Open*
Manuscript ID	bmjopen-2017-018097
Article Type:	Protocol
Date Submitted by the Author:	23-Jun-2017
Complete List of Authors:	Mordi, Natalie; University of Dundee Division of Cardiovascular and Diabetes Medicine<br>Mordi, Ify; University of Dundee Division of Cardiovascular and Diabetes Medicine<br>Singh, Jagdeep; University of Dundee Division of Cardiovascular and Diabetes Medicine<br>Baig, Fatima; University of Dundee Division of Cardiovascular and Diabetes Medicine<br>Choy, Anna-Maria; University of Dundee Division of Cardiovascular and Diabetes Medicine<br>McCrimmon, Rory; University of Dundee Division of Cardiovascular and Diabetes Medicine<br>Struthers, Allan; University of Dundee, Division of Cardiovascular and Diabetes Medicine<br>Lang, Chim C.; University of Dundee Division of Cardiovascular and Diabetes Medicine
**Primary Subject Heading**:	Cardiovascular medicine
Secondary Subject Heading:	Diabetes and endocrinology, Pharmacology and therapeutics, Research methods
Keywords:	Sodium-Glucose co-Transporter 2 (SGLT2) Inhibitors, Heart failure < CARDIOLOGY, Diabetes Mellitus, Natriuresis, Diuresis

SCHOLARONE™
Manuscripts

Research into the Renal and Cardiovascular Effect of Sodium-Glucose co-Transporter 2 (SGLT2) inhibition in combination with Loop Diuretics in Diabetic patients with Chronic Heart Failure (RECEDE-CHF): A Randomised Controlled Double-Blind Crossover Trial: Trial Rationale and Protocol

Natalie A. Mordi* **MRCP** n.a.mordi@dundee.ac.uk, **Ify R. Mordi MD MRCP** i.mordi@dundee.ac.uk, **Jagdeep S. S. Singh MRCP** j.x.singh@dundee.ac.uk, **Fatima Baig BSc.** f.z.baig@dundee.ac.uk, **AnnaMaria Choy FRCP FACC** a.choy@dundee.ac.uk, **Rory J. McCrimmon MD** r.mccrimmon@dundee.ac.uk, **Allan D. Struthers MD FRCP** a.d.struthers@dundee.ac.uk, **Chim C. Lang MD FACC** c.c.lang@dundee.ac.uk

***Correspondence:**

Dr Natalie A. Mordi

Division of Molecular and Clinical Medicine, Mailbox 2, Ninewells Hospital and Medical School, University of Dundee, Dundee DD1 9SY, UK

Phone: +44-1382-383013

Email: n.a.mordi@dundee.ac.uk

Short title: SGLT2 Inhibition in combination with diuretics in heart failure

Word Count: Abstract: 285. Excluding title page, abstract, references, figures and tables: 4627

Key words: Heart failure, diabetes mellitus, sodium-glucose co-transporter 2 (SGLT2) inhibitors, natriuresis, diuresis

Version 1.5. 23rd June 2017

For peer review only

ABSTRACT

Objectives: To assess the effect of Sodium-Glucose Linked Co-Transporter 2 (SGLT2) inhibitors when used in combination with a loop diuretic on diuresis and natriuresis when compared to placebo. We hypothesise that in those with Type 2 Diabetes (T2D) and Chronic Heart Failure (CHF), SGLT2 inhibition may augment the effects of loop diuretics.

Setting: The RECEDE-CHF trial is a single centre, randomised double-blind, placebo-controlled, crossover trial conducted in a secondary care setting within NHS Tayside, Scotland.

Participants: 34 eligible participants, aged between 18 to 80 years, with stable T2D and well-controlled CHF will be recruited. Participants will be excluded where there is a diagnosis of chronic liver disease, renal impairment or hypotension (systolic BP < 95 mmHg) at the screening visit.

Interventions: Renal physiological testing will be performed at two points (week 1 and week 6) on each arm to assess the effect of 25mg empagliflozin, on the primary and secondary outcomes, when compared to placebo. There will be a two-week washout period between each arm. Participants will be enrolled in the trial for a total period between 14 to 16 weeks.

Outcome measures: The primary outcome is to assess the effect of empagliflozin versus placebo on urine output. The secondary outcomes for this trial are to assess the effect of empagliflozin on

- urinary sodium excretion
- glomerular filtration rate

For peer review only - http://bmjopen.bmj.com/site/about/guidelines.xhtml

- creatinine
- protein/creatinine ratio
- albumin/creatinine ratio
- and on the renal biomarker, cystatin C when compared to placebo.

Conclusions: In this proof of concept trial, we hypothesise that the benefits of SGLT2 inhibitors extend beyond those of their metabolic (glycaemic parameters and weight loss) and haemodynamic parameters; that the effects of SGLT2 inhibitors as an osmotic diuretic and on natriuresis may underlie the cardiovascular and renal benefits demonstrated in the recent EMPA-REG study.

Trial registration: will be in place by the commencement of the trial.

For peer review only

For peer review only - http://bmjopen.bmj.com/site/about/guidelines.xhtml

ARTICLE SUMMARY

STRENGTHS AND LIMITATIONS

- **Original proof of concept study that aims to explore the effect on diuresis and natriuresis of SGLT2 inhibitors in combination with furosemide**
- **Randomised, double-blind crossover study design**
- **Small, single centre study**
- **High participant dropout rate has been factored into power calculations**
- **Aims to shed light on the mechanism of the cardiovascular benefits seen in EMPA-REG OUTCOME trial**

For peer review only

BACKGROUND

Chronic heart failure (CHF) and type 2 diabetes (T2D) frequently coexist. In population-based studies and in CHF trials, the prevalence of T2D among patients with symptomatic HF is estimated to be between 12% and 41%. [1] T2D has consistently been shown to be an independent predictor of increased morbidity and mortality in patients with CHF.[2]

For most patients**,** metformin is the first choice anti-diabetic drug in all T2D patients including those with coincidental HF.[3] However, metformin alone is often not enough to keep glycaemia under control and there is a frequent need for a second line anti-diabetic drug in HF patients. Sulphonylureas (SU) are commonly prescribed in T2D however they are associated with weight gain and hypoglycaemia, while there remain concerns that SUs may increase all-cause and cardiovascular (CV) mortality,[4]although this link is not fully established. Glitazones are contra-indicated in New York Heart Association (NYHA) III or IV HF, while their role in milder degrees of HF remains to a certain extent controversial with some observational studies indicating increased hospitalisation or readmission due to HF.[5] Insulin use has also been associated with increased mortality in patients with CHF.[6] More recent agents such as the DPP-IV inhibitors have also, disappointingly, failed to show cardiovascular benefit with some concerns raised following the publication of SAVOR-TIMI-53, that they increased HF hospitalisations, although these trials have only been of short duration[7] and may not apply to all DPP-IV inhibitors. Therefore, it can be concluded that therapeutic options in DM and HF limited due to a lack of evidence-based guidelines on the optimal management of such patients. Indeed, international guidelines recognize the evidence gap on the safety and efficacy of drugs used to treat DM in patients with HF as well as the need for agents that will both improve overall glycaemic control and HF outcomes.[8]

SGLT2 Inhibitors and HF

SGLT2 inhibitors are licensed for use in patients with T2D. These oral anti-diabetic agents achieve their effects by blocking the low affinity, high capacity Type 2 Sodium-

For peer review only

Glucose Linked Co-Transporter (SGLT2), predominantly found in the proximal convoluted tubules of the kidneys, thus causing glycosuria. The recent landmark EMPA-REG outcome study reported a striking 35% relative risk reduction in HF hospitalisations with empagliflozin may provide supportive evidence for beneficial effects of SGLT2 inhibition in the setting of CHF.[9] A recent analysis of the EMPA-REG study showed that empagliflozin reduced HF hospitalization and cardiovascular death, with a consistent benefit in patients with and without baseline HF.[10]

The effect of empagliflozin on HF hospitalization or CV death and on all-cause hospitalization was observed very early and was sustained throughout the trial. This suggests that the benefit was not driven by an effect on atherosclerosis. The mechanisms behind the effects of empagliflozin on HF and CV death are unknown. It has been hypothesised that the benefits of SGLT2 inhibitors extend beyond those of the glycaemic parameters of weight loss as promoted by glycosuria, but the effects of SGLT2 inhibitors on non-glycaemia parameters including blood pressure lowering as well as osmotic diuretic and natriuretic effects which may underlie the cardiovascular (and renal) benefits.[11,12]

Renal effects of SGLT2 inhibition and co-prescribing with Loop Diuretics

The renal effects of SGLT2 inhibitors are attracting much recent interest as they may confer renal protection.[13,14] There is data to support the potential for direct renoprotective actions arising from SGLT2 inhibition including actions to attenuate T1D associated hyperfiltration through an effect on tubulo-glomerular (TG) feedback which may have renal-protective effects by decreasing glomerular hydrostatic pressure.[13,14] SGLT2 inhibition has also been shown to attenuate tubular hypertrophy and reduce the tubular toxicity of glucose.[15] They may also have indirect renoprotective effects through its blood pressure lowering effects and glycaemia lowering effects which could decrease the renal inflammatory and fibrotic response by blocking glucose entry into the cell.[15] Consequently, there are now several on-going SGLT2 inhibitor renal outcome trials in T2D, including the CANVAS-R trial (clinicaltrials.gov identifier NCT01989754) and the CREDENCE trial (clinical trials.gov identifier NCT02065791). However, neither trial specifically looks at T2DM patients with CHF.

For peer review only - http://bmjopen.bmj.com/site/about/guidelines.xhtml

Studies relating to diuresis in the context of SGLT2 inhibitors are surprisingly sparse.[12] Previous studies with empagliflozin and canagliflozin have demonstrated a 24 hour urinary increase by 300 ml/day after day 1 of treatment but that the daily urinary volume returned to baseline after several weeks.[16,17] One Japanese case report however, in a non-diabetic patient, described successful treatment of fluid overload that was initially resistant to diuretic therapy, with 5 days of treatment of 50mg ipragliflozin.[18]

In post-hoc analysis of EMPA-REG OUTCOME, Fitchett et al. reported reduced use of furosemide in patients on the empagliflozin arm, suggesting that these patients reached a relative state of euvolaemia.[10,12] Heerspink et al. highlighted that, volume depletion and associated use of loop diuretic is long associated with a pre-renal cause of acute kidney injury, and a decrease in loop diuretic may also be relevant in light of the reductions in acute kidney injury, acute renal failure and chronic kidney disease progression endpoints.[12,19]

The renal effects of SGLT2 inhibitors in combination with furosemide in T2D with CHF are not known but given the relative frequency of both co-morbidities they are likely to be prescribed concurrently. This underscores the need for a trial to provide detailed acute and long-term information regarding the renal effects of SGLT2 inhibition in combination with loop diuretics, in T2D patients with stable CHF.

We hypothesize that SGLT2 inhibitors may be able to address the issue of diuretic resistance and may augment the diuretic effects of furosemide in patients with T2D and CHF.

We will recruit diabetic HF patients taking stable doses of furosemide, or, alternative loop diuretics, with eGFR greater than 45ml/min/1.73m^2. This trial will, with careful monitoring, begin the process of uncovering the unrealised potential of this new class of drug which, for the reasons outlined above, is poised to become the 2nd line anti-diabetic agent of choice in HF patients.

METHODS

Trial design

For peer review only

The RECEDE-CHF Trial is a single centre phase IV, randomised, double-blind, placebo-controlled, crossover, trial conducted in NHS Tayside, Scotland to compare the SGLT2 inhibitor empagliflozin 25mg, to placebo. Participants will be enrolled in the trial for a period of between 14 to 16 weeks. The trial design is summarised in **Figure 1 and Table 1.**

Figure 1: RECEDE-CHF Trial Design

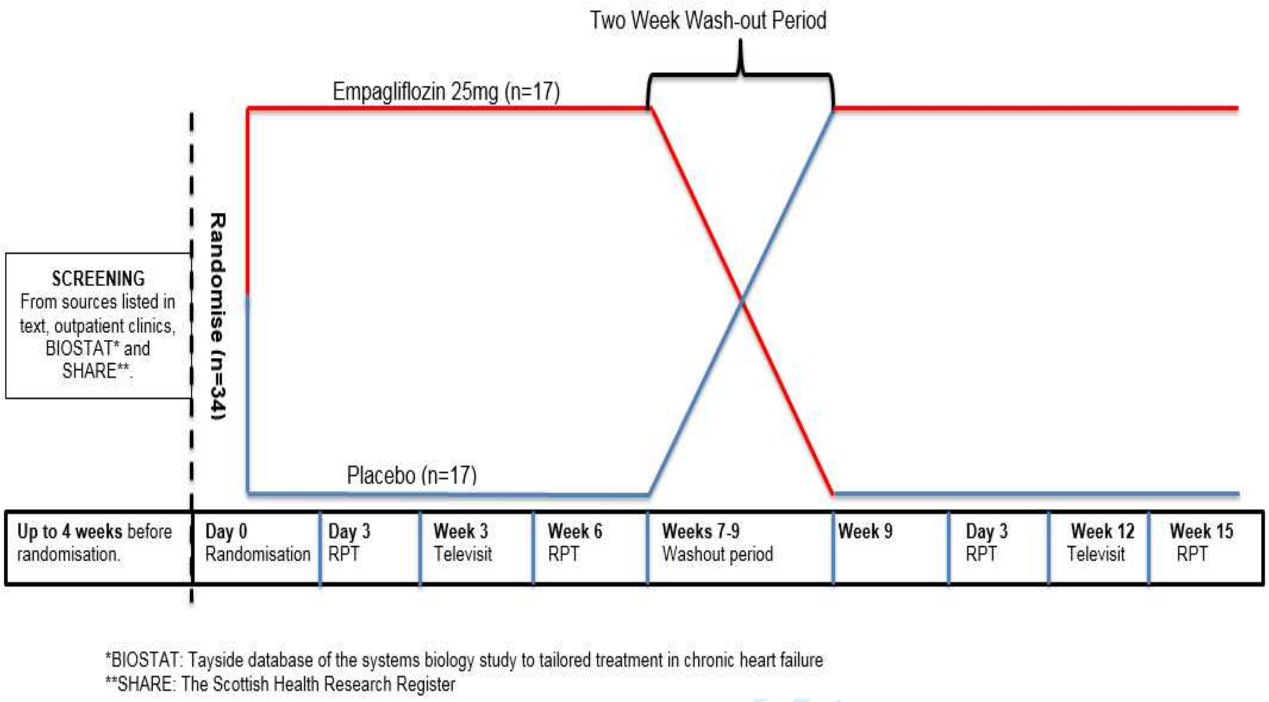

For peer review only

For peer review only - http://bmjopen.bmj.com/site/about/guidelines.xhtml

Table 1. RECEDE-CHF Trial Protocol

**Visit**	Visit 1* Screening	Visit 2* Baseline/ Randomisation	Visit 3	Visit 4 (Tele Visit)	Visit 5	Two week wash-out period	Visit 6	Visit 7	Visit 8 (Tele Visit)	Visit 9 (Final Visit)****
**Week**	Up to 4 weeks pre visit 2	**Day 0**	**Day 3** (+/-2 days)	**Week 3** (+/- 3 days)	**Week 6** (+/- 3 days)		**Week 9** (+/- 3 days)	**Week 9 + 3 days** (+/- 2 days)	**Week 12** (+/- 3 days)	**Week 15** (+/- 3 days)
**Informed Consent**	X									
**Inclusion/Exclusion Criteria**	X	X					X	X		
**Past Medical History**	X									
**Demographics**	X				X		X			X
**Vital Signs**	X	X	X		X		X	X		X
**Safety Bloods**	X	X	X		X		X	X		X
**Research Bloods**		X	X		X		X	X		X
**Genetic Blood Sample****		X								
**uPCR/uACR**		X	X		X		X	X		X
**Urine Pregnancy Test*****	X	X	X		X		X	X		X
**24 urinary collection**			X		X			X		X
**Renal Physiology Test**			X		X			X		X
**Drug Dispensing**		X					X			
**AE assessment**		X	X	X	X		X	X	X	X
**Record/Review Meds**	X	X	X	X	X		X	X	X	X
**Drug Compliance**			X	X	X			X	X	X

For peer review only - http://bmjopen.bmj.com/site/about/guidelines.xhtml

**Check**										

Visits 1 and Visits 2 combined into one visit where able *
Genetic Blood Sample** - only to be taken if participant consent given.
Urine Pregnancy Test*** - testing on females of childbearing potential or who do not abstain from sex or use effective contraception.
Final Visit**** - if the participant wishes to withdraw prematurely or at the PI's discretion, all study procedures will be conducted as though the final visit, if participant agrees.
uPCR/uACR urine protein/creatinine ratio, urine albumin/creatinine ratio

For peer review only

At the screening visit, following informed consent, an initial medical history and clinical examination will be performed and concomitant medication will be recorded. Participants will have bloods taken for safety analysis and vital signs will be checked to confirm eligibility prior to enrolment. An assessment of suitability of the trial for the potential participant will be undertaken by the principal investigator (PI) or medically qualified delegate.

Should the participant meet the inclusion criteria and have no exclusion criteria identified, they will return for the baseline/randomisation visit at the Clinical Research Centre (CRC), Ninewells Hospital, Dundee, within four weeks post screening visit. Where able the screening visit and randomisation visits will be combined.

At the randomisation visit participants will undergo safety blood tests, vital signs and study medication will be dispensed (either empagliflozin 25mg or placebo).

Participants will continue on study medication, empagliflozin 25mg or placebo, once daily, for a period of 6 weeks. Participants will be educated on the symptoms of hypoglycaemia and given a written action plan on how to manage it in the unlikely event that it occurs.

Participants will return to the CRC 3-days (±2 days) post-randomisation, for a study day where they will have safety and research bloods drawn, vital signs recorded and will undergo renal physiological tests (RPT). Further details of these RPT are described below.

Participants will then return again at week 6 for a study day where they will undergo RPT again, safety and research bloods will be drawn and vital signs recorded. Participants will terminate study drug, either empagliflozin 25mg or placebo, at this visit and will return to the CRC at the end of the two week wash out period (week 9).

At week 9, participants will have safety and research bloods drawn, vital signs recorded, new study medication dispensed. The RPT will then be repeated at the same intervals, at week 9 + 3 days (±2 days) and at week 14 for the final study day. Participants will then terminate study drug, either empagliflozin 25mg or placebo.

For peer review only - http://bmjopen.bmj.com/site/about/guidelines.xhtml

Study population

34 patients with underlying diabetes and well-controlled chronic heart failure (CHF) will be recruited from a range of sources. The local Tayside database of the systems biology study to tailored treatment in chronic heart failure (BIOSTAT) database consisting of around 1800 patients with HF who have previously consented to be approached for future research. The investigators may also recruit from NHS Tayside diabetes and/or heart failure clinics, SHARE The Scottish Health Research Register, where participants have pre-consented to be invited for research. It is anticipated it will take up to 18 months to recruit the 34 patients for randomisation with approximately 80-100 being consented into the screening trial. We anticipate a screen failure rate of 20% and a drop-out rate of 35% after recruitment based on previous heart failure studies within this research group and the high intensity of the Renal Physiology Test days which will occur on 4 occasions in total.

Eligibility

Patients will be eligible if they:

- Aged 18 to 80 years with previously diagnosed Type 2 Diabetes Mellitus.
- Are diagnosed with NYHA Functional class II-III HF with prior echocardiographic evidence of LVSD.
- On stable doses of furosemide, or alternative loop diuretic for one month
- Stable Type 2 Diabetes (HbA1c, in the last 3 months, of 10.0% ≥ and ≥ 6.5%)
- Point of Care BNP > 100 pg/ml
- eGFR ≥ 45 ml/min/1.73m^2
- Have stable HF symptoms for at least three months prior to consent
- On stable HF therapy for at least three months prior to consent
- Have not been hospitalised for HF for at least three months prior to consent

Patients will be excluded if they:

- A diagnosis of chronic liver disease and/or liver enzymes that are twice the upper limit of normal
- Systolic BP of <95mmHg at screening visit.

For peer review only - http://bmjopen.bmj.com/site/about/guidelines.xhtml

- eGFR < 45 ml/min/1.73m^2
- Patients on thiazide diuretics
- Malignancy (receiving active treatment) or other life threatening disease.
- Pregnant or lactating women
- Patients with difficulty in micturition e.g. severe prostate enlargement
- Patients who have participated in any other clinical trial of an investigational medicinal product within 30days
- Patients who are unable to give informed consent
- Any other reason considered by the physician to be inappropriate for inclusion.

Randomisation and treatment allocation

After successful screening for eligibility and safety, participants will be randomised to either empagliflozin 25mg/placebo or placebo/empagliflozin 25 mg in a double blind fashion in this crossover study.

The double blind medication (empagliflozin or placebo) will be prepared, packaged and labelled by our onsite clinical trials pharmaceutical manufacturer. Randomisation will be carried out by our dedicated clinical trials pharmacy using block randomisation. They will use a validated randomisation program and will securely backup both the randomisation seed and the randomisation allocation and have it available in the onsite 24-h emergency unblinding facility.

Participants will be given blinded medication. At the randomisation visit they will be dosed with either empagliflozin 25mg (6 weeks) or matched placebo (6 weeks) to continue once daily, with a 2-week washout period between each arm. Participants and their bloods (including urea & electrolytes, liver function tests, and full blood count) will be monitored as per trial schedule and medication stopped if concerns arise. If the trial drug needs to be stopped due to intolerance or adverse events, they will remain in the trial in order to do an “intention to treat” analysis.

Trial outcomes

For peer review only - http://bmjopen.bmj.com/site/about/guidelines.xhtml

The primary aim/objectives will be to assess whether empagliflozin (SGLT2 Inhibitor) can augment the diuretic effects of loop diuretics in diabetic patients with mild CHF with left ventricular systolic dysfunction (LVSD), as measured by urinary volume, compared to placebo.

The secondary aims/objectives are to assess the effect of empagliflozin (SGLT2 inhibitor) on natriuresis when used with loop diuretics in diabetic patients with mild CHF with LVSD as measured by urinary sodium excretion, to measure the safety of add-on SGLT2 inhibitor therapy versus placebo on top of loop diuretics as measured by serum creatinine and eGFR, to assess effects of empagliflozin on protein/creatinine ratio, albumin/creatinine ratio and on the renal biomarker, cystatin C.[20]

Renal Physiology Tests

Patients will attend the Clinical Research Centre (CRC), Ninewells, Dundee on 4 separate study days (2 while in each arm of the trial). On each study day, patients will present themselves to our CRC, following an overnight fast. Two days before presenting to the CRC, patients will be required to follow a 2gram sodium and 2L fluid/day controlled diet. A 24-hour urinary collection will be requested the day prior to presentation at the CRC for urinary volume and sodium. Patients will be asked to take their morning usual medications except their investigational medicinal product (empagliflozin or placebo) and furosemide (or equivalent loop diuretic).

An intravenous (IV) cannula will be placed in each arm for subsequent infusion and blood sampling. Bloods will be drawn for measurement of plasma NT-proBNP and cystatin C using standard protocols. A 15 ml/kg oral water load will be administered over a 15 min period. Thereafter, at 30 min intervals, patients will be requested to void urine until the end of the study period. The volume of urine passed will be measured and an aliquot stored for later analysis. On each occasion, the volume of urine passed will be measured, and an equal volume of water given to drink. In this way, a steady state diuresis will be established over approximately over 3 hours, avoiding the need for catheterisation. The last 30 min urinary collection during this stabilization period will be taken as baseline. At + 150 minutes, each patient will receive an oral tablet of either

For peer review only - http://bmjopen.bmj.com/site/about/guidelines.xhtml

empagliflozin 25mg or placebo. At + 210 minutes, patients will be given a bolus of IV furosemide at half their total daily dose.

Heart rate and blood pressure will be displayed continuously on an ECG oscilloscope and the blood pressure measured every 60 min. Venous blood will be obtained at the midpoint of each clearance period for measurement of serum sodium, osmolality and creatinine. **Figure 2** outlines the protocol for the RPT test day.

For peer review only

For peer review only - http://bmjopen.bmj.com/site/about/guidelines.xhtml

Figure 2: Protocol for Renal Physiology Test Days

e.g. Time	Interval	Urine		Blood	
0730	- 30				Arrives fasted. 2 x cannula sited
0745	- 15				15mls/kg oral water load over 15 minutes
0800	0				
0830	+ 30	Void urine. Water intake equal to urine volume.			
0900	+ 60	Void urine. Water intake equal to urine volume			
0930	+ 90	Void urine. Water intake equal to urine volume.			
0945	+ 105			Bloods (serum B1) for U&Es & osmolality	
1000	+ 120	Void urine. Water intake equal to urine volume. Collect urine (urine B1) for volume, sodium, creatinine & osmolality.	B1 (90-120 mins)		
1015	+ 135			Bloods (serum B2) for U&Es & osmolality	
1030	+ 150	Void urine. Water intake equal to urine volume. Collect urine (urine B2) for volume, sodium, creatinine & osmolality	B2 (120-150 mins)		IMP administered: Oral tablet (empagliflozin 20mg or placebo)
1045	+ 165		D1 (150-180 mins)	Bloods (serum D1) for U&Es & osmolality	
1100	+ 180	Void urine. Water intake equal to urine volume.			

For peer review only - http://bmjopen.bmj.com/site/about/guidelines.xhtml

		Collect urine for volume, sodium, creatinine & osmolality			
1115	+ 195		D2 (180-210 mins)	Bloods (serum D2) for U&Es & osmolality	
1130	+ 210	Void urine. Water intake equal to urine volume. Collect urine for volume, sodium, creatinine & osmolality			IV Frusemide administered: half participant's screening dose
1145	+ 225		F1 (210 – 240 mins)	Bloods (serum F1) for U&Es & osmolality	
1200	+ 240	Void urine. Water intake equal to urine volume. Collect urine for volume, sodium, creatinine & osmolality			
1215	+ 255		F2 (240-270 mins)	Bloods (serum F2) for U&Es & osmolality	
1230	+ 270	Collect urine for volume, sodium, creatinine & osmolality.			**END OF STUDY PERIOD**

For peer review only

For peer review only - http://bmjopen.bmj.com/site/about/guidelines.xhtml

IV furosemide will be administered to eliminate variable furosemide gut absorption and for practical reasons to complete the study within the set time frame due to the reduced time for the peak onset of diuretic effect with IV administration.

Data on the effect of SLGT2 inhibitors and the participant's usual oral diuretic regime will be gained by the collection of 24 urinary samples on the days before the RPT.[21]

Tolerability

We will monitor for tolerability of the study medication. One of the inevitable side effects of empagliflozin and other SGLT2 inhibitors with the promotion of glycosuria is urinary tract infections. This typically presents itself as genital mycotic infection, typically Candida albicans, which is usually straightforward to treat. However, if patients were to present with infection unresponsive to standard treatment then the trial drug would be discontinued. They will also be warned of the side effects including hypoglycaemia with a written action plan on how to recognise and treat in the unlikely event of a hypoglycaemic episode

Sample size and power calculations

Power calculations were performed by the University of Dundee's Senior Medical Statistician and are based on our previous data in patients with CHF[21] as well as more contemporary data.[22] The sample size is based on the mean furosemide-induced urinary volume and sodium excretion of 920 mls/hr (SD=250) and 300 µmol/min (SD=60) respectively. A 20% increase is expected in these parameters following SGLT2 inhibition. This increase in urinary volume is based on published percentage increases in urinary volume in T2D patients that range from 11%-33% depending on the dose of the SGLT2 inhibitor as shown by List and colleagues[23] in the available data with dapagliflozin (dapagliflozin 2.5 mg: 11% increase in 24-hr urine volume; dapagliflozin 5 mg: 22% 11% increase in 24-hr urine volume; dapagliflozin 10 mg: 24% 11% increase in 24-hr urine volume; dapagliflozin 20 mg: 27% 11% increase in 24-hr urine volume and dapagliflozin 50 mg: 33% 11% increase in 24-hr urine volume). There is no data of SGLT inhibition together with loop diuretics. As described previously the Japanese case report of a diuretic resistant HF patient in whom fluid overload was successfully treated

For peer review only - http://bmjopen.bmj.com/site/about/guidelines.xhtml

with the SGLT2 inhibitor, ipragliflozin, where a striking 50% increase in urinary volume (following treatment with 50 mg daily of oral ipragliflozin for 5 days) was described.[18] Based on the above, it was determined that a 20% increase in furosemide induced increase in urinary volume and sodium excretion will be reasonable.

With an alpha 0·05 and power of 90%, 22 participants per arm are required, assuming 35% drop out. Since this trial is using an AB/BA crossover design, a total of 34 participants will be required, as each participant will be exposed to both arms of the trial. The rationale for the high dropout rate, is due to the high intensity renal physiological tests that will occur on 4 occasions for the participants, from 0730 hrs to 1400 hrs we feel that anything less than a 35% drop out rate would be conservative.

Data Collection

The data will be collected by the PI or delegate on a paper case report form (CRF) with subsequent transcription to an electronic CRF. Electronic storage will be in an encrypted form on a password protected device. The medical notes will act as source data for past medical history and blood results. Any data relating to general medical history will be documented in the notes.

DISCUSSION

SGLT2 inhibition is an innovative strategy for the management of T2D, where historically, anti-hyperglycemic interventions have focused on restoring B-cell activity, insulin sensitivity, or tissue glucose uptake to normalise plasma glucose levels.[12]

In this proof of concept trial, we hypothesise that the benefits of SGLT2 inhibitors extend beyond those of their metabolic (glycaemic parameters and weight loss) and haemodynamic parameters; that the effects of SGLT2 inhibitors as an osmotic diuretic and on natriuresis may underlie the cardiovascular and renal benefits.

Whilst the encouraging renal and cardiovascular outcomes demonstrated in EMPA-REG might be explained by the modest but cumulative effect of blood pressure lowering (mean reduction in systolic BP ~ 3 mmHg), HbA1c reduction (0.3%), and weight reduction (~ 1kg), it is difficult to ignore the possibility that an additional mechanism may

For peer review only - http://bmjopen.bmj.com/site/about/guidelines.xhtml

also be at work.[19,24] For example, Mudaliar et al hypothesise that empagliflozin may also improve renal fuel energetics and efficiency, providing more energy efficient oxygen consumption and thereby potentially less hypoxic stress on the diabetic heart and kidney.[24]

The SGLT2 inhibitors may also have indirect renoprotective effects through its blood pressure lowering effects and glycaemia lowering effects which could decrease the renal inflammatory and fibrotic response by blocking glucose entry into the cell.[15] Consequently, there are now several on-going SGLT2 inhibitor renal outcome trials in T2D, including the CANVAS-R trial (clinicaltrials.gov identifier NCT01989754) and the CREDENCE trial (clinical trials.gov identifier NCT02065791). However, neither trial specifically looks at T2D patients with CHF.

We have also described that SGLT2 inhibitors may augment the effect of loop diuretics. It is noteworthy that osmotic diuretics such as mannitol have been used alone or in combination with loop diuretics such as furosemide to promote diuresis in patients undergoing intra-cranial surgery[25] and in the postoperative period to prevent acute kidney injury.[26] In CHF, mannitol was reported to promote effective diuresis in a single centre study in the US.[27] Importantly, in all these settings, mannitol, which is a potent osmotic diuretic when used in combination with furosemide, was shown to be safe and did not result in renal failure or electrolyte disturbances.

By detailing urinary volumes and sodium excretion via renal physiological tests at two points 3 days and 6 weeks into the investigational medicinal product, the trial's primary aims to assess the change to these markers representative of diuresis in patients on empagliflozin and when compared to placebo. The effects on natriuresis, proteinuria, albuminuria, cystatin C will also be studied as will the safety of add-on SGLT2 inhibition versus placebo.

LIMITATIONS

Whilst power calculations have been conducted to calculate the sample size, an obvious limitation is that this is a small single centre trial. The proposed trial will require

For peer review only - http://bmjopen.bmj.com/site/about/guidelines.xhtml

participants on four occasions to undergo detailed renal physiological tests, (from 0730-1400) as such a dropout rate has been factored in of 35%.

Heart failure is a dynamic disease; its natural history is one of flux as the patient's intravascular volume changes, their loop diuretic requirement may also fluctuate. However, as stipulated on the inclusion criteria, we will be taking patients with heart failure (BNP > 100 pg/ml) with evidence of previous LVSD, but with stable symptoms and medications for 3 months, who have not had a hospital admission for heart failure within this same time frame.

CONCLUSION

Whilst the osmotic diuretic hypothesis[11] is frequently discussed in relation to the renal and cardiovascular outcomes with SGLT2 inhibition, literature on the effect of SGLT2 inhibitors on diuresis is currently limited. At time of writing, no studies have been performed to assess the effect of loop diuretics when used in combination with SGLT2 inhibitors. This proof of concept trial will aim to shed light on the mechanism of the cardiovascular and renal outcomes demonstrated in the recent EMPA-REG study by documenting the influence of the SGLT2 inhibitors when used in combination with a loop diuretic on urinary volumes and natriuresis when compared to placebo.

Further intent of this proposed study is to obtain data that might be relevant to the design of future studies in which SGLT2 inhibitors may be considered as an adjunctive agent to loop diuretics in patients who experience diuretic resistance. Although data is currently limited, since HF and T2D are frequent co-morbidities, it is probable that physicians may find patients requiring both SGLT2 inhibitor therapy and furosemide concurrently. Only by studying this, can these fundamental issues be addressed and, perhaps, inspire a change in practise.

List of abbreviations

T2D: type 2 diabetes, HF: heart failure, CHF: chronic heart failure, EASD: European Association for the Study of Diabetes, ADA: American Diabetes Association, SU: sulphonylureas, CV: cardiovascular, NYHA: New York Heart Association, DPPIV inhibitors: Dipeptidyl peptidase-4, SGLT2 inhibitors: Sodium-Glucose Linked co-

For peer review only - http://bmjopen.bmj.com/site/about/guidelines.xhtml

Transporter 2 inhibitors, LVSD: left ventricular systolic dysfunction, LV: left ventricular, TG: tubulo-glomerular, eGFR: estimated glomerular filtration rate, CRC: Clinical Research Centre, Ninewells Hospital, Dundee, UK, RPT: renal physiological tests, EFSD: European Foundation for the Study of Diabetes, PI: principal investigator, BIOSTAT: A systems BIOlogy Study to TAilored Treatment in Chronic Heart Failure, SHARE: The Scottish Health Research Register, CI: Chief Investigator

DECLARATIONS

Ethics approval and consent to participate

This protocol has been reviewed by the East of Scotland Research Ethics Service. Reference number 16/ES/0137.

Consent for publication

Not applicable.

Availability of data and material

Not applicable.

Competing interests

ADS has received research support from Astra Zeneca, as well as lecture and consulting fees also from Astra Zeneca. CCL has received research support and consulting fees from Novartis, research support, lecture fees, and consulting fees from AstraZeneca, lecture fees from Merck Sharp & Dohme, and research support from Pfizer and Sanofi. AMC has received research support from St Jude Medical, lecture fees for Daiichi Sankyo, travel support from Boston Scientific, Biotronik, Medtronic, St Jude Medical. RJM has served on advisory boards for Novo Nordisk, Sanofi, Lilly. Rest of the authors declare no competing interests.

Funding

The RECEDE-CHF trial is funded by the British Heart Foundation (BHF grant number: 807998). NAM is a BHF funded clinical research fellow.

Authors' contributions

For peer review only

NAM participated in the design of the study, data collection and drafting the manuscript. IM and JSS participated in the design of the study and reviewing the manuscript. FB and AMC read and approved the final manuscript. RJM and ADS participated in study design and coordination as well as reading and approving the final manuscript. CCL conceived the study, participated in its design and helped draft the manuscript.

Acknowledgements

Our gratitude to all members of staff within the department of Division of Cardiovascular and Diabetes Medicine at the University of Dundee, and the team at the Clinical Research Centre, University of Dundee, Ninewells Hospital and Medical School, UK.

REFERENCES

1. MacDonald MR, Petrie MC, Hawkins NM, et al. Diabetes, left ventricular systolic dysfunction, and chronic heart failure. *Eur Heart J*. 2008;29(10):1224-1240. doi:10.1093/eurheartj/ehn156.

2. Murcia AM, Hennekens CH, Lamas GA, et al. Impact of diabetes on mortality in patients with myocardial infarction and left ventricular dysfunction. *Arch Intern Med*. 2004;164(20):2273-2279. doi:10.1001/archinte.164.20.2273.

3. Krum H, Jelinek M V, Stewart S, et al. *2011 Update to National Heart Foundation of Australia and Cardiac Society of Australia and New Zealand Guidelines for the Prevention, Detection and Management of Chronic Heart Failure in Australia, 2006.* Vol 194.; 2011. http://eutils.ncbi.nlm.nih.gov/entrez/eutils/elink.fcgi?dbfrom=pubmed&id=21495941&retmode=ref&cmd=prlinks.

4. Forst T, Hanefeld M, Jacob S, et al. Association of sulphonylurea treatment with all-cause and cardiovascular mortality: a systematic review and meta-analysis of observational studies. *Diab Vasc Dis Res*. 2013;10(4):302-314. doi:10.1177/1479164112465442.

5. Eurich DT, McAlister FA, Blackburn DF, et al. Benefits and harms of antidiabetic agents in patients with diabetes and heart failure: systematic review. *BMJ*. 2007;335(7618):497. doi:10.1136/bmj.39314.620174.80.

6. Smooke S, Horwich TB, Fonarow GC. Insulin-treated diabetes is associated with a marked increase in mortality in patients with advanced heart failure. *Am Heart J*. 2005;149(1):168-174. doi:10.1016/j.ahj.2004.07.005.

7. Scirica BM, Bhatt DL, Braunwald E, et al. Saxagliptin and cardiovascular outcomes in patients with type 2 diabetes mellitus. *N Engl J Med*.

For peer review only - http://bmjopen.bmj.com/site/about/guidelines.xhtml

2013;369(14):1317-1326. doi:10.1056/NEJMoa1307684.

8. Ryden L, Grant PJ, Anker SD, et al. ESC guidelines on diabetes, pre-diabetes, and cardiovascular diseases developed in collaboration with the EASD. *Eur Heart J*. 2013;34(39):3035-3087. doi:10.1093/eurheartj/eht108.

9. Zinman B, Wanner C, Lachin JM, et al. Empagliflozin, Cardiovascular Outcomes, and Mortality in Type 2 Diabetes. *N Engl J Med*. 2015;373(22):2117-2128. doi:10.1056/NEJMoa1504720.

10. Fitchett D, Zinman B, Wanner C, et al. Heart failure outcomes with empagliflozin in patients with type 2 diabetes at high cardiovascular risk: results of the EMPA-REG OUTCOME(R) trial. *Eur Heart J*. 2016;37(19):1526-1534. doi:10.1093/eurheartj/ehv728.

11. McMurray J. EMPA-REG - The "diuretic hypothesis." *J Diabetes Complications*. 2016;30(1):3-4. doi:10.1016/j.jdiacomp.2015.10.012.

12. Heerspink HJL, Perkins BA, Fitchett DH, Husain M, Cherney DZI. Sodium Glucose Cotransporter 2 Inhibitors in the Treatment of Diabetes Mellitus: Cardiovascular and Kidney Effects, Potential Mechanisms, and Clinical Applications. *Circulation*. 2016;134(10):752-772. doi:10.1161/CIRCULATIONAHA.116.021887.

13. Skrtic M, Cherney DZI. Sodium-glucose cotransporter-2 inhibition and the potential for renal protection in diabetic nephropathy. *Curr Opin Nephrol Hypertens*. 2015;24(1):96-103 8p. doi:10.1097/MNH.0000000000000084.

14. Cherney DZI, Perkins BA, Soleymanlou N, et al. Renal hemodynamic effect of sodium-glucose cotransporter 2 inhibition in patients with type 1 diabetes mellitus. *Circulation*. 2014;129(5):587-597. doi:10.1161/CIRCULATIONAHA.113.005081.

15. Thomas MC. Renal effects of dapagliflozin in patients with type 2 diabetes. *Ther Adv Endocrinol Metab*. 2014;5(3):53-61. doi:10.1177/2042018814544153.

16. Sha S Heise T, Natarajan J, Farrell K, Wang SS, Sica D, Rothenberg P, Plum-Morschel L PD. Effect of the sodium glucose co-transporter 2 inhibitor canagliflozin on plasma volume in patients with type 2 diabetes mellitus. *Diabetes Obes Metab*. 2014;16(11):1087. http://ovidsp.ovid.com/ovidweb.cgi?T=JS&PAGE=reference&D=cctr&NEWS=N&AN=CN-01115818.

17. Heise T, Macha S, Hantel S, Pinnetti S, Seman L, Woerle HJ. Safety, tolerability, pharmacokinetics and pharmacodynamics following 4 weeks' treatment with empagliflozin once daily in patients with type 2 diabetes. *Diabetes, Obes Metab*. 2013;15:613-621. doi:10.1111/dom.12073.

18. Sairaku A, Nakano Y, Kihara Y. Increased urine output by ipragliflozin in a non-diabetic patient with a diuretic-resistant heart failure. *Int J Cardiol*. 2015;180:42-43. doi:10.1016/j.ijcard.2014.11.138.

19. Wanner C, Inzucchi SE, Lachin JM, et al. Empagliflozin and Progression of

For peer review only

Kidney Disease in Type 2 Diabetes. *N Engl J Med*. 2016;375(4):323-334. doi:10.1056/NEJMoa1515920.

20. Veldhuisen DJ Van, Ruilope LM, Maisel AS, Damman K. Clinical update Biomarkers of renal injury and function : diagnostic , prognostic and therapeutic implications in heart failure. 2016:2577-2585. doi:10.1093/eurheartj/ehv588.

21. Lang C, Choy A, Rahman A, Struthers A. Renal effects of low dose prazosin in patients with congestive heart failure. *Eur Heart J*. 1993;14(9):1245-1252. http://onlinelibrary.wiley.com/o/cochrane/clcentral/articles/819/CN-00096819/frame.html.

22. Goldsmith S, Gilbertson D, Mackedanz S, Swan S. Renal effects of conivaptan, furosemide, and the combination in patients with chronic heart failure. *J Card Fail*. 2011;17(12):982-989. doi:10.1016/j.cardfail.2011.08.012.

23. List JF, Woo V, Morales E, Tang W, Fiedorek FT. Sodium-glucose cotransport inhibition with dapagliflozin in type 2 diabetes. *Diabetes Care*. 2009;32(4):650-657. doi:10.2337/dc08-1863.

24. Mudaliar S, Alloju S, Henry RR. Can a shift in fuel energetics explain the beneficial cardiorenal outcomes in the EMPA-REG OUTCOME study? a unifying hypothesis. *Diabetes Care*. 2016;39(7):1115-1122. doi:10.2337/dc16-0542.

25. Bebawy JF, Ramaiah VK, Zeeni C, Hemmer LB, Koht A, Gupta DK. The effect of furosemide on intravascular volume status and electrolytes in patients receiving mannitol: an intraoperative safety analysis. *J Neurosurg Anesthesiol*. 2013;25(1):51-54. doi:10.1097/ANA.0b013e318269c335.

26. Sirivella S, Gielchinsky I, Parsonnet V. Mannitol, furosemide, and dopamine infusion in postoperative renal failure complicating cardiac surgery. *Ann Thorac Surg*. 2000;69(2):501-506. doi:10.1016/S0003-4975(99)01298-9.

27. Turagam MK, Velagapudi P, Kalra AS, Ramalingam VS, Sinnakirouchenan R, Holley JL. Outcomes of furosemide-mannitol infusion in hospitalized patients with heart failure: an observational single-center cohort study of 122 patients. *Int J Cardiol*. 2011;151(2):232-234. doi:10.1016/j.ijcard.2011.06.044.

For peer review only

STANDARD PROTOCOL ITEMS: RECOMMENDATIONS FOR INTERVENTIONAL TRIALS

SPIRIT 2013 Checklist: Recommended items to address in a clinical trial protocol and related documents*

For peer review only

Section/item	Item No	Description	Addressed on page number
**Administrative information**			
Title	1	Descriptive title identifying the study design, population, interventions, and, if applicable, trial acronym	1
Trial registration	2a	Trial identifier and registry name. If not yet registered, name of intended registry	3
	2b	All items from the World Health Organization Trial Registration Data Set	
Protocol version	3	Date and version identifier	1
Funding	4	Sources and types of financial, material, and other support	23
Roles and responsibilities	5a	Names, affiliations, and roles of protocol contributors	23
	5b	Name and contact information for the trial sponsor	1
	5c	Role of study sponsor and funders, if any, in study design; collection, management, analysis, and interpretation of data; writing of the report; and the decision to submit the report for publication, including whether they will have ultimate authority over any of these activities	23
	5d	Composition, roles, and responsibilities of the coordinating centre, steering committee, endpoint adjudication committee, data management team, and other individuals or groups overseeing the trial, if applicable (see Item 21a for data monitoring committee)	23

For peer review only - http://bmjopen.bmj.com/site/about/guidelines.xhtml

**Introduction**			
Background and rationale	6a	Description of research question and justification for undertaking the trial, including summary of relevant studies (published and unpublished) examining benefits and harms for each intervention	____5-7_______
	6b	Explanation for choice of comparators	_____7_______
Objectives	7	Specific objectives or hypotheses	___14-15_______
Trial design	8	Description of trial design including type of trial (eg, parallel group, crossover, factorial, single group), allocation ratio, and framework (eg, superiority, equivalence, noninferiority, exploratory)	______7-8______
**Methods: Participants, interventions, and outcomes**			
Study setting	9	Description of study settings (eg, community clinic, academic hospital) and list of countries where data will be collected. Reference to where list of study sites can be obtained	______2_______
Eligibility criteria	10	Inclusion and exclusion criteria for participants. If applicable, eligibility criteria for study centres and individuals who will perform the interventions (eg, surgeons, psychotherapists)	_____13-14_____
Interventions	11a	Interventions for each group with sufficient detail to allow replication, including how and when they will be administered	___2, 15-17_____
	11b	Criteria for discontinuing or modifying allocated interventions for a given trial participant (eg, drug dose change in response to harms, participant request, or improving/worsening disease)	_____19________
	11c	Strategies to improve adherence to intervention protocols, and any procedures for monitoring adherence (eg, drug tablet return, laboratory tests)	______10_______
	11d	Relevant concomitant care and interventions that are permitted or prohibited during the trial	______19_______
Outcomes	12	Primary, secondary, and other outcomes, including the specific measurement variable (eg, systolic blood pressure), analysis metric (eg, change from baseline, final value, time to event), method of aggregation (eg, median, proportion), and time point for each outcome. Explanation of the clinical relevance of chosen efficacy and harm outcomes is strongly recommended	____2, 15_______

For peer review only - http://bmjopen.bmj.com/site/about/guidelines.xhtml

Participant timeline	13	Time schedule of enrolment, interventions (including any run-ins and washouts), assessments, and visits for participants. A schematic diagram is highly recommended (see Figure)	_______10______
Sample size	14	Estimated number of participants needed to achieve study objectives and how it was determined, including clinical and statistical assumptions supporting any sample size calculations	_____19________
Recruitment	15	Strategies for achieving adequate participant enrolment to reach target sample size	______19_______
**Methods: Assignment of interventions (for controlled trials)**			
Allocation:			
Sequence generation	16a	Method of generating the allocation sequence (eg, computer-generated random numbers), and list of any factors for stratification. To reduce predictability of a random sequence, details of any planned restriction (eg, blocking) should be provided in a separate document that is unavailable to those who enrol participants or assign interventions	______14_______
Allocation concealment mechanism	16b	Mechanism of implementing the allocation sequence (eg, central telephone; sequentially numbered, opaque, sealed envelopes), describing any steps to conceal the sequence until interventions are assigned	_____14________
Implementation	16c	Who will generate the allocation sequence, who will enrol participants, and who will assign participants to interventions	___13-14_______
Blinding (masking)	17a	Who will be blinded after assignment to interventions (eg, trial participants, care providers, outcome assessors, data analysts), and how	______14_______
	17b	If blinded, circumstances under which unblinding is permissible, and procedure for revealing a participant's allocated intervention during the trial	______14_______
**Methods: Data collection, management, and analysis**			
Data collection methods	18a	Plans for assessment and collection of outcome, baseline, and other trial data, including any related processes to promote data quality (eg, duplicate measurements, training of assessors) and a description of study instruments (eg, questionnaires, laboratory tests) along with their reliability and validity, if known. Reference to where data collection forms can be found, if not in the protocol	_____20________

For peer review only - http://bmjopen.bmj.com/site/about/guidelines.xhtml

	18b	Plans to promote participant retention and complete follow-up, including list of any outcome data to be collected for participants who discontinue or deviate from intervention protocols	_____________
Data management	19	Plans for data entry, coding, security, and storage, including any related processes to promote data quality (eg, double data entry; range checks for data values). Reference to where details of data management procedures can be found, if not in the protocol	_____20________
Statistical methods	20a	Statistical methods for analysing primary and secondary outcomes. Reference to where other details of the statistical analysis plan can be found, if not in the protocol	_not included ___
	20b	Methods for any additional analyses (eg, subgroup and adjusted analyses)	_____________
	20c	Definition of analysis population relating to protocol non-adherence (eg, as randomised analysis), and any statistical methods to handle missing data (eg, multiple imputation)	_____________
**Methods: Monitoring**			
Data monitoring	21a	Composition of data monitoring committee (DMC); summary of its role and reporting structure; statement of whether it is independent from the sponsor and competing interests; and reference to where further details about its charter can be found, if not in the protocol. Alternatively, an explanation of why a DMC is not needed	____n/a________
	21b	Description of any interim analyses and stopping guidelines, including who will have access to these interim results and make the final decision to terminate the trial	_____________
Harms	22	Plans for collecting, assessing, reporting, and managing solicited and spontaneously reported adverse events and other unintended effects of trial interventions or trial conduct	______20_______
Auditing	23	Frequency and procedures for auditing trial conduct, if any, and whether the process will be independent from investigators and the sponsor	_____________
**Ethics and dissemination**			
Research ethics approval	24	Plans for seeking research ethics committee/institutional review board (REC/IRB) approval	_____22-23_____

For peer review only - http://bmjopen.bmj.com/site/about/guidelines.xhtml

Protocol amendments	25	Plans for communicating important protocol modifications (eg, changes to eligibility criteria, outcomes, analyses) to relevant parties (eg, investigators, REC/IRBs, trial participants, trial registries, journals, regulators)	______________
Consent or assent	26a	Who will obtain informed consent or assent from potential trial participants or authorised surrogates, and how (see Item 32)	_________12_____
	26b	Additional consent provisions for collection and use of participant data and biological specimens in ancillary studies, if applicable	______________
Confidentiality	27	How personal information about potential and enrolled participants will be collected, shared, and maintained in order to protect confidentiality before, during, and after the trial	______________
Declaration of interests	28	Financial and other competing interests for principal investigators for the overall trial and each study site	_____23________
Access to data	29	Statement of who will have access to the final trial dataset, and disclosure of contractual agreements that limit such access for investigators	______________
Ancillary and post-trial care	30	Provisions, if any, for ancillary and post-trial care, and for compensation to those who suffer harm from trial participation	______________
Dissemination policy	31a	Plans for investigators and sponsor to communicate trial results to participants, healthcare professionals, the public, and other relevant groups (eg, via publication, reporting in results databases, or other data sharing arrangements), including any publication restrictions	______________
	31b	Authorship eligibility guidelines and any intended use of professional writers	______________
	31c	Plans, if any, for granting public access to the full protocol, participant-level dataset, and statistical code	______________
**Appendices**			
Informed consent materials	32	Model consent form and other related documentation given to participants and authorised surrogates	__not included___
Biological specimens	33	Plans for collection, laboratory evaluation, and storage of biological specimens for genetic or molecular analysis in the current trial and for future use in ancillary studies, if applicable	______________

For peer review only - http://bmjopen.bmj.com/site/about/guidelines.xhtml

*It is strongly recommended that this checklist be read in conjunction with the SPIRIT 2013 Explanation & Elaboration for important clarification on the items. Amendments to the protocol should be tracked and dated. The SPIRIT checklist is copyrighted by the SPIRIT Group under the Creative Commons "Attribution-NonCommercial-NoDerivs 3.0 Unported" license.

For peer review only

For peer review only - http://bmjopen.bmj.com/site/about/guidelines.xhtml

The Renal and Cardiovascular Effect of Sodium-Glucose co-Transporter 2 (SGLT2) inhibition in combination with Loop Diuretics in Diabetic patients with Chronic Heart Failure (RECEDE-CHF): Protocol for a Randomised Controlled Double-Blind Crossover Trial.

Journal:	*BMJ Open*
Manuscript ID	bmjopen-2017-018097.R1
Article Type:	Protocol
Date Submitted by the Author:	11-Jul-2017
Complete List of Authors:	Mordi, Natalie; University of Dundee Division of Cardiovascular and Diabetes Medicine<br>Mordi, Ify; University of Dundee Division of Cardiovascular and Diabetes Medicine<br>Singh, Jagdeep; University of Dundee Division of Cardiovascular and Diabetes Medicine<br>Baig, Fatima; University of Dundee Division of Cardiovascular and Diabetes Medicine, Division of Cardiovascular and Diabetes Medicine<br>Choy, Anna-Maria; University of Dundee Division of Cardiovascular and Diabetes Medicine<br>McCrimmon, Rory; University of Dundee Division of Cardiovascular and Diabetes Medicine<br>Struthers, Allan; University of Dundee, Division of Cardiovascular and Diabetes Medicine<br>Lang, Chim C.; University of Dundee Division of Cardiovascular and Diabetes Medicine
**Primary Subject Heading**:	Cardiovascular medicine
Secondary Subject Heading:	Diabetes and endocrinology, Pharmacology and therapeutics, Research methods
Keywords:	Sodium-Glucose co-Transporter 2 (SGLT2) Inhibitors, Heart failure < CARDIOLOGY, Diabetes Mellitus, Natriuresis, Diuresis

SCHOLARONE™ Manuscripts

The Renal and Cardiovascular Effect of Sodium-Glucose co-Transporter 2 (SGLT2) inhibition in combination with Loop Diuretics in Diabetic patients with Chronic Heart Failure (RECEDE-CHF): Protocol for a Randomised Controlled Double-Blind Crossover Trial.

Natalie A. Mordi* **MRCP** n.a.mordi@dundee.ac.uk, **Ify R. Mordi MD MRCP** i.mordi@dundee.ac.uk, **Jagdeep S. S. Singh MRCP** j.x.singh@dundee.ac.uk, **Fatima Baig BSc.** f.z.baig@dundee.ac.uk, **AnnaMaria Choy FRCP FACC** a.choy@dundee.ac.uk, **Rory J. McCrimmon MD** r.mccrimmon@dundee.ac.uk, **Allan D. Struthers MD FRCP** a.d.struthers@dundee.ac.uk, **Chim C. Lang MD FACC** c.c.lang@dundee.ac.uk

***Correspondence:**

Dr Natalie A. Mordi

Division of Molecular and Clinical Medicine, Mailbox 2, Ninewells Hospital and Medical School, University of Dundee, Dundee DD1 9SY, UK

Phone: +44-1382-383013

Email: n.a.mordi@dundee.ac.uk

Short title: SGLT2 Inhibition in combination with diuretics in heart failure

Word Count: Abstract: 298. Excluding title page, abstract, figures and references: 5075

Key words: Heart failure, diabetes mellitus, sodium-glucose co-transporter 2 (SGLT2) inhibitors, natriuresis, diuresis

Version 2.0. 9th July 2017

For peer review only - http://bmjopen.bmj.com/site/about/guidelines.xhtml

ABSTRACT

Introduction: Type 2 diabetes (T2D) and heart failure (HF) are a frequent combination, where treatment options remain limited. There has been increasing interest around the sodium-glucose co-transporter 2 (SGLT2) inhibitors and their use in patients with HF. Data on the effect of SGLT2 inhibitor use with diuretics is limited. We hypothesise that SGLT2 inhibition may augment the effects of loop diuretics.

Methods and Analysis: To assess the effect of SGLT2 inhibitors when used in combination with a loop diuretic, the RECEDE-CHF trial is a single centre, randomised double-blind, placebo-controlled, crossover trial conducted in a secondary care setting within NHS Tayside, Scotland.

34 eligible participants, aged between 18 to 80 years, with stable T2D and CHF will be recruited. Renal physiological testing will be performed at two points (week 1 and week 6) on each arm to assess the effect of 25mg empagliflozin, on the primary and secondary outcomes. Participants will be enrolled in the trial for a total period between 14 to 16 weeks.

The primary outcome will assess the effect of empagliflozin versus placebo on urine output. The secondary outcomes are to assess the effect of empagliflozin on glomerular filtration rate, cystatin C, urinary sodium excretion, urinary protein/creatinine ratio, and urinary albumin/creatinine ratio when compared to placebo.
We hypothesise that the benefits of SGLT2 inhibitors extend beyond those of their metabolic (glycaemic parameters and weight loss) and haemodynamic parameters; that the effects of SGLT2 inhibitors as an osmotic diuretic and on natriuresis may underlie the cardiovascular and renal benefits demonstrated in the recent EMPA-REG study.
Ethics and Dissemination: Ethics approval was obtained by the East of Scotland Research Ethics Service. Results of the trial will be submitted for publication in a peer-reviewed journal.

Registration Details: Prospective registration will be obtained via clinicaltrials.gov: before the enrolment of patients.

For peer review only

ARTICLE SUMMARY

STRENGTHS AND LIMITATIONS

- **Original proof of concept study that aims to explore the effect on diuresis and natriuresis of SGLT2 inhibitors in combination with furosemide**
- **Randomised, double-blind crossover study design**
- **Small, single centre study**
- **High participant dropout rate has been factored into power calculations**
- **Aims to shed light on the mechanism of the cardiovascular benefits seen in EMPA-REG OUTCOME trial**

For peer review only

BACKGROUND

Chronic heart failure (CHF) and type 2 diabetes (T2D) frequently coexist. In population-based studies and in CHF trials, the prevalence of T2D among patients with symptomatic HF is estimated to be between 12% and 41%. [1] T2D has consistently been shown to be an independent predictor of increased morbidity and mortality in patients with CHF.[2]

For most patients, metformin is the first choice anti-diabetic drug in all T2D patients including those with coincidental HF.[3] However, metformin alone is often not enough to keep glycaemia under control and there is a frequent need for a second line anti-diabetic drug in HF patients. Sulphonylureas (SU) are commonly prescribed in T2D however they are associated with weight gain and hypoglycaemia, while there remain concerns that SUs may increase all-cause and cardiovascular (CV) mortality,[4]although this link is not fully established. Glitazones are contra-indicated in New York Heart Association (NYHA) III or IV HF, while their role in milder degrees of HF remains to a certain extent controversial with some observational studies indicating increased hospitalisation or readmission due to HF.[5] Insulin use has also been associated with increased mortality in patients with CHF.[6] More recent agents such as the DPP-IV inhibitors have also, disappointingly, failed to show cardiovascular benefit with some concerns raised following the publication of SAVOR-TIMI-53, that they increased HF hospitalisations, although these trials have only been of short duration[7] and may not apply to all DPP-IV inhibitors. Therefore, it can be concluded that therapeutic options in DM and HF limited due to a lack of evidence-based guidelines on the optimal management of such patients. Indeed, international guidelines recognize the evidence gap on the safety and efficacy of drugs used to treat DM in patients with HF as well as the need for agents that will both improve overall glycaemic control and HF outcomes.[8]

SGLT2 Inhibitors and HF

SGLT2 inhibitors are licensed for use in patients with T2D. These oral anti-diabetic agents achieve their effects by blocking the low affinity, high capacity Type 2 Sodium-Glucose Linked Co-Transporter (SGLT2), predominantly found in the proximal convoluted tubules of the kidneys, thus causing glycosuria. The recent landmark

For peer review only

EMPA-REG outcome study reported a striking 35% relative risk reduction in HF hospitalisations with empagliflozin may provide supportive evidence for beneficial effects of SGLT2 inhibition in the setting of CHF.[9] A recent analysis of the EMPA-REG study showed that empagliflozin reduced HF hospitalization and cardiovascular death, with a consistent benefit in patients with and without baseline HF.[10]

The effect of empagliflozin on HF hospitalization or CV death and on all-cause hospitalization was observed very early and was sustained throughout the trial. This suggests that the benefit was not driven by an effect on atherosclerosis. The mechanisms behind the effects of empagliflozin on HF and CV death are unknown. It has been hypothesised that the benefits of SGLT2 inhibitors extend beyond those of the glycaemic parameters of weight loss as promoted by glycosuria, but the effects of SGLT2 inhibitors on non-glycaemia parameters including blood pressure lowering as well as osmotic diuretic and natriuretic effects which may underlie the cardiovascular (and renal) benefits.[11,12]

Renal effects of SGLT2 inhibition and co-prescribing with Loop Diuretics

The renal effects of SGLT2 inhibitors are attracting much recent interest as they may confer renal protection.[13,14] There is data to support the potential for direct renoprotective actions arising from SGLT2 inhibition including actions to attenuate T1D associated hyperfiltration through an effect on tubulo-glomerular (TG) feedback which may have renal-protective effects by decreasing glomerular hydrostatic pressure.[13,14] SGLT2 inhibition has also been shown to attenuate tubular hypertrophy and reduce the tubular toxicity of glucose.[15] They may also have indirect renoprotective effects through its blood pressure lowering effects and glycaemia lowering effects which could decrease the renal inflammatory and fibrotic response by blocking glucose entry into the cell.[15] Consequently, there are now several on-going SGLT2 inhibitor renal outcome trials in T2D, including the CANVAS-R trial (clinicaltrials.gov identifier NCT01989754) and the CREDENCE trial (clinical trials.gov identifier NCT02065791). However, neither trial specifically looks at T2DM patients with CHF.

Studies relating to diuresis in the context of SGLT2 inhibitors are surprisingly sparse.[12] Previous studies with empagliflozin and canagliflozin have demonstrated a 24 hour

For peer review only

urinary increase by 300 ml/day after day 1 of treatment but that the daily urinary volume returned to baseline after several weeks.[16,17] One Japanese case report however, in a non-diabetic patient, described successful treatment of fluid overload that was initially resistant to diuretic therapy, with 5 days of treatment of 50mg ipragliflozin.[18]

In post-hoc analysis of EMPA-REG OUTCOME, Fitchett et al. reported reduced use of furosemide in patients on the empagliflozin arm, suggesting that these patients reached a relative state of euvolaemia.[10,12] Heerspink et al. highlighted that, volume depletion and associated use of loop diuretic is long associated with a pre-renal cause of acute kidney injury, and a decrease in loop diuretic may also be relevant in light of the reductions in acute kidney injury, acute renal failure and chronic kidney disease progression endpoints.[12,19]

The renal effects of SGLT2 inhibitors in combination with furosemide in T2D with CHF are not known but given the relative frequency of both co-morbidities they are likely to be prescribed concurrently. This underscores the need for a trial to provide detailed acute and long-term information regarding the renal effects of SGLT2 inhibition in combination with loop diuretics, in T2D patients with stable CHF.

We hypothesize that SGLT2 inhibitors may be able to address the issue of diuretic resistance and may augment the diuretic effects of furosemide in patients with T2D and CHF.

We will recruit diabetic HF patients taking stable doses of furosemide, or, alternative loop diuretics, with eGFR greater than 45ml/min/1.73m^2. This trial will, with careful monitoring, begin the process of uncovering the unrealised potential of this new class of drug which, for the reasons outlined above, is poised to become the 2nd line anti-diabetic agent of choice in HF patients.

METHODS: PARTICIPANTS, INTERVENTIONS, AND OUTCOMES

Trial design

For peer review only

The RECEDE-CHF Trial is a single centre phase IV, randomised, double-blind, placebo-controlled, crossover, trial conducted in NHS Tayside, Scotland to compare the SGLT2 inhibitor empagliflozin 25mg, to placebo. Participants will be enrolled in the trial for a period of between 14 to 16 weeks. The trial design is summarised in **Figure 1 and Table 1.**

For peer review only

Table 1. RECEDE-CHF Trial Protocol

**Visit**	Visit 1* Screening	Visit 2* Baseline/ Randomisation	Visit 3	Visit 4 (Tele Visit)	Visit 5	Two week wash-out period	Visit 6	Visit 7	Visit 8 (Tele Visit)	Visit 9 (Final Visit)****
**Week**	Up to 4 weeks pre visit 2	**Day 0**	**Day 3** (+/-2 days)	**Week 3** (+/- 3 days)	**Week 6** (+/- 3 days)		**Week 9** (+/- 3 days)	**Week 9 + 3 days** (+/- 2 days)	**Week 12** (+/- 3 days)	**Week 15** (+/- 3 days)
**Informed Consent**	X									
**Inclusion/Exclusion Criteria**	X	X					X	X		
**Past Medical History**	X									
**Demographics**	X				X		X			X
**Vital Signs**	X	X	X		X		X	X		X
**Safety Bloods**	X	X	X		X		X	X		X
**Research Bloods**		X	X		X		X	X		X
**Genetic Blood Sample****		X								
**uPCR/uACR**		X	X		X		X	X		X
**Urine Pregnancy Test*****	X	X	X		X		X	X		X
**24 urinary collection**			X		X			X		X
**Renal Physiology Test**			X		X			X		X
**Drug Dispensing**		X					X			
**AE assessment**		X	X	X	X		X	X	X	X
**Record/Review Meds**	X	X	X	X	X		X	X	X	X
**Drug Compliance**			X	X	X			X	X	X

For peer review only

Check										

Visits 1 and Visits 2 combined into one visit where able *
Genetic Blood Sample** - only to be taken if participant consent given.
Urine Pregnancy Test*** - testing on females of childbearing potential or who do not abstain from sex or use effective contraception.
Final Visit**** - if the participant wishes to withdraw prematurely or at the PI's discretion, all study procedures will be conducted as though the final visit, if participant agrees.
uPCR/uACR urine protein/creatinine ratio, urine albumin/creatinine ratio

For peer review only

At the screening visit, following informed consent, an initial medical history and clinical examination will be performed and concomitant medication will be recorded. Participants will have bloods taken for safety analysis and vital signs will be checked to confirm eligibility prior to enrolment. An assessment of suitability of the trial for the potential participant will be undertaken by the principal investigator (PI) or medically qualified delegate.

Should the participant meet the inclusion criteria and have no exclusion criteria identified, they will return for the baseline/randomisation visit at the Clinical Research Centre (CRC), Ninewells Hospital, Dundee, within four weeks post screening visit. Where able the screening visit and randomisation visits will be combined.

At the randomisation visit participants will undergo safety blood tests, vital signs and study medication will be dispensed (either empagliflozin 25mg or placebo).

Participants will continue on study medication, empagliflozin 25mg or placebo, once daily, for a period of 6 weeks. Participants will be educated on the symptoms of hypoglycaemia and given a written action plan on how to manage it in the unlikely event that it occurs.

Participants will return to the CRC 3-days (±2 days) post-randomisation, for a study day where they will have safety and research bloods drawn, vital signs recorded and will undergo renal physiological tests (RPT). Further details of these RPT are described below.

Participants will then return again at week 6 for a study day where they will undergo RPT again, safety and research bloods will be drawn and vital signs recorded. Participants will terminate study drug, either empagliflozin 25mg or placebo, at this visit and will return to the CRC at the end of the two week wash out period (week 9).

At week 9, participants will have safety and research bloods drawn, vital signs recorded, new study medication dispensed. The RPT will then be repeated at the same intervals, at week 9 + 3 days (±2 days) and at week 14 for the final study day. Participants will then terminate study drug, either empagliflozin 25mg or placebo.

For peer review only

Study population

34 patients with underlying diabetes and well-controlled chronic heart failure (CHF) will be recruited from a range of sources. The local Tayside database of the systems biology study to tailored treatment in chronic heart failure (BIOSTAT) database consisting of around 1800 patients with HF who have previously consented to be approached for future research. The investigators may also recruit from NHS Tayside diabetes and/or heart failure clinics, SHARE The Scottish Health Research Register, where participants have pre-consented to be invited for research. It is anticipated it will take up to 18 months to recruit the 34 patients for randomisation with approximately 80-100 being consented into the screening trial. We anticipate a screen failure rate of 20% and a drop-out rate of 35% after recruitment based on previous heart failure studies within this research group and the high intensity of the Renal Physiology Test days which will occur on 4 occasions in total.

Eligibility

Patients will be eligible if they:

- Aged 18 to 80 years with previously diagnosed Type 2 Diabetes Mellitus.
- Are diagnosed with NYHA Functional class II-III HF with prior echocardiographic evidence of LVSD.
- On stable doses of furosemide, or alternative loop diuretic for one month
- Stable Type 2 Diabetes (HbA1c, in the last 3 months, of 10.0% ≥ and ≥ 6.5%)
- Point of Care BNP > 100 pg/ml
- eGFR ≥ 45 ml/min/1.73m^2
- Have stable HF symptoms for at least three months prior to consent
- On stable HF therapy for at least three months prior to consent
- Have not been hospitalised for HF for at least three months prior to consent

Patients will be excluded if they:

- A diagnosis of chronic liver disease and/or liver enzymes that are twice the upper limit of normal
- Systolic BP of <95mmHg at screening visit.

For peer review only - http://bmjopen.bmj.com/site/about/guidelines.xhtml

- eGFR < 45 ml/min/1.73m^2
- Patients on thiazide diuretics
- Malignancy (receiving active treatment) or other life threatening disease.
- Pregnant or lactating women
- Patients with difficulty in micturition e.g. severe prostate enlargement
- Patients who have participated in any other clinical trial of an investigational medicinal product within 30days
- Patients who are unable to give informed consent
- Any other reason considered by the physician to be inappropriate for inclusion.

Randomisation and treatment allocation

After successful screening for eligibility and safety, participants will be randomised to either empagliflozin 25mg/placebo or placebo/empagliflozin 25 mg in a double blind fashion in this crossover study.

The double blind medication (empagliflozin or placebo) will be prepared, packaged and labelled by our onsite clinical trials pharmaceutical manufacturer. Randomisation will be carried out by our dedicated clinical trials pharmacy using block randomisation. They will use a validated randomisation program and will securely backup both the randomisation seed and the randomisation allocation and have it available in the onsite 24-h emergency unblinding facility.

Participants will be given blinded medication. At the randomisation visit they will be dosed with either empagliflozin 25mg (6 weeks) or matched placebo (6 weeks) to continue once daily, with a 2-week washout period between each arm. Participants and their bloods (including urea & electrolytes, liver function tests, and full blood count) will be monitored as per trial schedule and medication stopped if concerns arise. If the trial drug needs to be stopped due to intolerance or adverse events, they will remain in the trial in order to do an “intention to treat” analysis.

Trial outcomes

For peer review only - http://bmjopen.bmj.com/site/about/guidelines.xhtml

The primary aim/objectives will be to assess whether empagliflozin (SGLT2 Inhibitor) can augment the diuretic effects of loop diuretics in diabetic patients with mild CHF with left ventricular systolic dysfunction (LVSD), as measured by urinary volume, compared to placebo.

The secondary aims/objectives are to assess the effect of empagliflozin (SGLT2 inhibitor) on natriuresis when used with loop diuretics in diabetic patients with mild CHF with LVSD as measured by urinary sodium excretion, to measure the safety of add-on SGLT2 inhibitor therapy versus placebo on top of loop diuretics as measured by serum creatinine and eGFR, to assess effects of empagliflozin on protein/creatinine ratio, albumin/creatinine ratio and on the renal biomarker, cystatin C.[20]

Renal Physiology Tests

Patients will attend the Clinical Research Centre (CRC), Ninewells, Dundee on 4 separate study days (2 while in each arm of the trial). On each study day, patients will present themselves to our CRC, following an overnight fast. Two days before presenting to the CRC, patients will be required to follow a 2gram sodium and 2L fluid/day controlled diet. A 24-hour urinary collection will be requested the day prior to presentation at the CRC for urinary volume and sodium. Patients will be asked to take their morning usual medications except their investigational medicinal product (empagliflozin or placebo) and furosemide (or equivalent loop diuretic).

An intravenous (IV) cannula will be placed in each arm for subsequent infusion and blood sampling. Bloods will be drawn for measurement of plasma NT-proBNP and cystatin C using standard protocols. A 15 ml/kg oral water load will be administered over a 15 min period. Thereafter, at 30 min intervals, patients will be requested to void urine until the end of the study period. The volume of urine passed will be measured and an aliquot stored for later analysis. On each occasion, the volume of urine passed will be measured, and an equal volume of water given to drink. In this way, a steady state diuresis will be established over approximately over 3 hours, avoiding the need for catheterisation. The last 30 min urinary collection during this stabilization period will be taken as baseline. At + 150 minutes, each patient will receive an oral tablet of either

For peer review only - http://bmjopen.bmj.com/site/about/guidelines.xhtml

empagliflozin 25mg or placebo. At + 210 minutes, patients will be given a bolus of IV furosemide at half their total daily dose.

Heart rate and blood pressure will be displayed continuously on an ECG oscilloscope and the blood pressure measured every 60 min. Venous blood will be obtained at the midpoint of each clearance period for measurement of serum sodium, osmolality and creatinine. **Figure 2** outlines the protocol for the RPT test day.

IV furosemide will be administered to eliminate variable furosemide gut absorption and for practical reasons to complete the study within the set time frame due to the reduced time for the peak onset of diuretic effect with IV administration.

Data on the effect of SLGT2 inhibitors and the participant's usual oral diuretic regime will be gained by the collection of 24 urinary samples on the days before the RPT.[21]

Tolerability

We will monitor for tolerability of the study medication. One of the inevitable side effects of empagliflozin and other SGLT2 inhibitors with the promotion of glycosuria is urinary tract infections. This typically presents itself as genital mycotic infection, typically Candida albicans, which is usually straightforward to treat. However, if patients were to present with infection unresponsive to standard treatment then the trial drug would be discontinued. They will also be warned of the side effects including hypoglycaemia with a written action plan on how to recognise and treat in the unlikely event of a hypoglycaemic episode

Sample size and power calculations

Power calculations were performed by the University of Dundee's Senior Medical Statistician and are based on our previous data in patients with CHF[21] as well as more contemporary data.[22] The sample size is based on the mean furosemide-induced urinary volume and sodium excretion of 920 mls/hr (SD=250) and 300 µmol/min (SD=60) respectively. A 20% increase is expected in these parameters following SGLT2 inhibition. This increase in urinary volume is based on published percentage increases in urinary volume in T2D patients that range from 11%-33% depending on the dose of

For peer review only - http://bmjopen.bmj.com/site/about/guidelines.xhtml

the SGLT2 inhibitor as shown by List and colleagues[23] in the available data with dapagliflozin (dapagliflozin 2.5 mg: 11% increase in 24-hr urine volume; dapagliflozin 5 mg: 22% 11% increase in 24-hr urine volume; dapagliflozin 10 mg: 24% 11% increase in 24-hr urine volume; dapagliflozin 20 mg: 27% 11% increase in 24-hr urine volume and dapagliflozin 50 mg: 33% 11% increase in 24-hr urine volume). There is no data of SGLT inhibition together with loop diuretics. As described previously the Japanese case report of a diuretic resistant HF patient in whom fluid overload was successfully treated with the SGLT2 inhibitor, ipragliflozin, where a striking 50% increase in urinary volume (following treatment with 50 mg daily of oral ipragliflozin for 5 days) was described.[18] Based on the above, it was determined that a 20% increase in furosemide induced increase in urinary volume and sodium excretion will be reasonable.

With an alpha 0·05 and power of 90%, 22 participants per arm are required, assuming 35% drop out. Since this trial is using an AB/BA crossover design, a total of 34 participants will be required, as each participant will be exposed to both arms of the trial. The rationale for the high dropout rate, is due to the high intensity renal physiological tests that will occur on 4 occasions for the participants, from 0730 hrs to 1400 hrs we feel that anything less than a 35% drop out rate would be conservative.

METHODS: DATA COLLECTION, MANAGEMENT, AND ANALYSIS

Data Collection

The data will be collected by the PI or delegate on a paper case report form (CRF) with subsequent transcription to an electronic CRF. Electronic storage will be in an encrypted form on a password protected device. The medical notes will act as source data for past medical history and blood results. Any data relating to general medical history will be documented in the notes.

Statistical Plan

Participants will be allocated to treatment groups at random. Descriptive statistics will be calculated for all data and reported as mean (SD) for continuous data and N (%) for categorical data. For statistical evaluation of repeated measurements, analysis of

For peer review only - http://bmjopen.bmj.com/site/about/guidelines.xhtml

variance will be used. P<0.05 will be taken as the level of statistical significance. The study is powered based on the primary outcome. In order to examine differences in patient characteristics between study groups, between-group comparisons will be assessed using independent t-tests or analysis of variance (ANOVA). Within-group comparisons will be conducted using paired sample t-tests or repeated measures ANOVA if data meet parametric assumptions.

METHODS: MONITORING

Data Monitoring

A Data Monitoring Committee is not considered necessary as this is a relatively small trial. Close supervision of the PI/Clinical Research Fellow will be conducted by an experienced Chief Investigator supported by a senior trial manager from the Tayside Clinical Trials Unit (TCTU).

The purposes of trial monitoring are to verify that the rights and well-being of human subjects are protected, the reported trial data are accurate, complete and verifiable from source documents and the conduct of the trial is in compliance with the currently approved protocol/amendment(s), with Good Clinical Practice (GCP), and with the applicable regulatory requirement(s). The Sponsor will determine the appropriate extent and nature of monitoring for the trial and will appoint appropriately qualified and trained monitors. The monitor will communicate any monitoring findings to both the CI and PI and the Sponsor.

Identifying, Recording and Reporting Adverse Events

Participants should be instructed to contact a member of the trial team at any time after consenting to join the trial if any of the above symptoms develop. All reported adverse events (AEs) that occur after joining the trial will be recorded in detail in the CRF AE log. In the case of an AE, the Investigator should initiate the appropriate treatment according to their medical judgement. Participants with AEs present at the last visit must be followed up until resolution of the event.

For peer review only

The CI or delegate will ask about the occurrence of AEs and hospitalisations at every visit during the trial. AEs will be recorded on the AE Log in the CRF. Serious adverse events (SAEs) will be submitted on an SAE form to the TASC Pharmacovigilance Section within 24 hours of becoming aware of the SAE. SAEs will be initially assessed for causality and expectedness by the Investigator. The Sponsor will make the definitive assessment on expectedness. The evaluation of expectedness will be made based on the knowledge of the reaction and the relevant product information (Summary of Product Characteristics).

Confidentiality

All laboratory specimens, evaluation forms, reports, and other records will be identified in a manner designed to maintain participant confidentiality. All records will be kept in a secure storage area with limited access to trial staff only. Clinical information will not be released without the written permission of the participant, except as necessary for monitoring and auditing by the Sponsor, its designee or Regulatory Authorities. The CI and trial staff will not disclose or use for any purpose other than performance of the trial, any data, record, or other unpublished, confidential information disclosed to those individuals for the purpose of the trial. Prior written agreement from the Sponsor or its designee will be obtained for the disclosure of any said confidential information to other parties.

ETHICS AND DISSEMINATION

Ethics approval was obtained by the East of Scotland Research Ethics Service. This trial has been funded by the British Heart Foundation who have peer reviewed the grant application. Additional peer review of the protocol occurs via the Sponsorship Committee.

The clinical trial report will be used for publication and presentation at scientific meetings. Trial investigators have the right to publish orally or in writing the results of the trial.

Prospective registration will be obtained via clinicaltrials.gov before the enrolment of patients.

For peer review only

Should any protocol modifications arise, these will be decided by the CI on discussion with the PI, which would will be escalated to the Trial Sponsor, who will decide if further notification to the relevant authorities is required.

Access to data

Ownership of the data arising from this trial resides with the trial team and their respective employers. On completion of the trial, the trial data will be analysed and tabulated, and a clinical trial report will be prepared.

DISCUSSION

SGLT2 inhibition is an innovative strategy for the management of T2D, where historically, anti-hyperglycemic interventions have focused on restoring B-cell activity, insulin sensitivity, or tissue glucose uptake to normalise plasma glucose levels.[12]

In this proof of concept trial, we hypothesise that the benefits of SGLT2 inhibitors extend beyond those of their metabolic (glycaemic parameters and weight loss) and haemodynamic parameters; that the effects of SGLT2 inhibitors as an osmotic diuretic and on natriuresis may underlie the cardiovascular and renal benefits.

Whilst the encouraging renal and cardiovascular outcomes demonstrated in EMPA-REG might be explained by the modest but cumulative effect of blood pressure lowering (mean reduction in systolic BP ~ 3 mmHg), HbA1c reduction (0.3%), and weight reduction (~ 1kg), it is difficult to ignore the possibility that an additional mechanism may also be at work.[19,24] For example, Mudaliar et al hypothesise that empagliflozin may also improve renal fuel energetics and efficiency, providing more energy efficient oxygen consumption and thereby potentially less hypoxic stress on the diabetic heart and kidney.[24]

The SGLT2 inhibitors may also have indirect renoprotective effects through its blood pressure lowering effects and glycaemia lowering effects which could decrease the renal inflammatory and fibrotic response by blocking glucose entry into the cell.[15] Consequently, there are now several on-going SGLT2 inhibitor renal outcome trials in T2D, including the CANVAS-R trial (clinicaltrials.gov identifier NCT01989754) and the

For peer review only - http://bmjopen.bmj.com/site/about/guidelines.xhtml

CREDENCE trial (clinical trials.gov identifier NCT02065791). However, neither trial specifically looks at T2D patients with CHF.

We have also described that SGLT2 inhibitors may augment the effect of loop diuretics. It is noteworthy that osmotic diuretics such as mannitol have been used alone or in combination with loop diuretics such as furosemide to promote diuresis in patients undergoing intra-cranial surgery[25] and in the postoperative period to prevent acute kidney injury.[26] In CHF, mannitol was reported to promote effective diuresis in a single centre study in the US.[27] Importantly, in all these settings, mannitol, which is a potent osmotic diuretic when used in combination with furosemide, was shown to be safe and did not result in renal failure or electrolyte disturbances.

By detailing urinary volumes and sodium excretion via renal physiological tests at two points 3 days and 6 weeks into the investigational medicinal product, the trial's primary aims to assess the change to these markers representative of diuresis in patients on empagliflozin and when compared to placebo. The effects on natriuresis, proteinuria, albuminuria, cystatin C will also be studied as will the safety of add-on SGLT2 inhibition versus placebo.

LIMITATIONS

Whilst power calculations have been conducted to calculate the sample size, an obvious limitation is that this is a small single centre trial. The proposed trial will require participants on four occasions to undergo detailed renal physiological tests, (from 0730-1400) as such a dropout rate has been factored in of 35%.

Heart failure is a dynamic disease; its natural history is one of flux as the patient's intravascular volume changes, their loop diuretic requirement may also fluctuate. However, as stipulated on the inclusion criteria, we will be taking patients with heart failure with evidence of previous LVSD, but with stable symptoms and medications for 3 months, who have not had a hospital admission for heart failure within this same time frame.

CONCLUSION

For peer review only - http://bmjopen.bmj.com/site/about/guidelines.xhtml

Whilst the osmotic diuretic hypothesis[11] is frequently discussed in relation to the renal and cardiovascular outcomes with SGLT2 inhibition, literature on the effect of SGLT2 inhibitors on diuresis is currently limited. At time of writing, no studies have been performed to assess the effect of loop diuretics when used in combination with SGLT2 inhibitors. This proof of concept trial will aim to shed light on the mechanism of the cardiovascular and renal outcomes demonstrated in the recent EMPA-REG study by documenting the influence of the SGLT2 inhibitors when used in combination with a loop diuretic on urinary volumes and natriuresis when compared to placebo.

Further intent of this proposed study is to obtain data that might be relevant to the design of future studies in which SGLT2 inhibitors may be considered as an adjunctive agent to loop diuretics in patients who experience diuretic resistance. Although data is currently limited, since HF and T2D are frequent co-morbidities, it is probable that physicians may find patients requiring both SGLT2 inhibitor therapy and furosemide concurrently. Only by studying this, can these fundamental issues be addressed and, perhaps, inspire a change in practise.

List of abbreviations

T2D: type 2 diabetes, HF: heart failure, CHF: chronic heart failure, EASD: European Association for the Study of Diabetes, ADA: American Diabetes Association, SU: sulphonylureas, CV: cardiovascular, NYHA: New York Heart Association, DPPIV inhibitors: Dipeptidyl peptidase-4, SGLT2 inhibitors: Sodium-Glucose Linked co-Transporter 2 inhibitors, LVSD: left ventricular systolic dysfunction, LV: left ventricular, TG: tubulo-glomerular, eGFR: estimated glomerular filtration rate, CRC: Clinical Research Centre, Ninewells Hospital, Dundee, UK, RPT: renal physiological tests, EFSD: European Foundation for the Study of Diabetes, PI: principal investigator, BIOSTAT: A systems BIOlogy Study to TAilored Treatment in Chronic Heart Failure, SHARE: The Scottish Health Research Register, CI: Chief Investigator, GCP: Good Clinical Practice, SD: Standard Deviation, ANOVA: analysis of variance, TCTU: Tayside Clinical Trials Unit, AEs: adverse events, SAEs: Serious adverse events, TASC: Tayside Medical Science Centre, SmPC: Summary of Product Characteristics.

For peer review only - http://bmjopen.bmj.com/site/about/guidelines.xhtml

DECLARATIONS

Ethics approval and consent to participate

This protocol has been reviewed by the East of Scotland Research Ethics Service. Reference number 16/ES/0137.

Consent for publication

Not applicable.

Availability of data and material

Not applicable.

Competing interests

ADS has received research support from Astra Zeneca, as well as lecture and consulting fees also from Astra Zeneca. CCL has received research support and consulting fees from Novartis, research support, lecture fees, and consulting fees from AstraZeneca, lecture fees from Merck Sharp & Dohme, and research support from Pfizer and Sanofi. AMC has received research support from St Jude Medical, lecture fees for Daiichi Sankyo, travel support from Boston Scientific, Biotronik, Medtronic, St Jude Medical. RJM has served on advisory boards for Novo Nordisk, Sanofi, Lilly. Rest of the authors declare no competing interests.

Funding

The RECEDE-CHF trial is funded by the British Heart Foundation (BHF grant number: 807998). NAM is a BHF funded clinical research fellow.

Authors' contributions

NAM participated in the design of the study, data collection and drafting the manuscript. IM and JSS participated in the design of the study and reviewing the manuscript. FB and AMC read and approved the final manuscript. RJM and ADS participated in study design and coordination as well as reading and approving the final manuscript. CCL conceived the study, participated in its design and helped draft the manuscript.

For peer review only

Acknowledgements

Our gratitude to all members of staff within the department of Division of Cardiovascular and Diabetes Medicine at the University of Dundee, and the team at the Clinical Research Centre, University of Dundee, Ninewells Hospital and Medical School, UK.

REFERENCES

1. MacDonald MR, Petrie MC, Hawkins NM, et al. Diabetes, left ventricular systolic dysfunction, and chronic heart failure. *Eur Heart J*. 2008;29(10):1224-1240. doi:10.1093/eurheartj/ehn156.
2. Murcia AM, Hennekens CH, Lamas GA, et al. Impact of diabetes on mortality in patients with myocardial infarction and left ventricular dysfunction. *Arch Intern Med*. 2004;164(20):2273-2279. doi:10.1001/archinte.164.20.2273.
3. Krum H, Jelinek M V, Stewart S, et al. *2011 Update to National Heart Foundation of Australia and Cardiac Society of Australia and New Zealand Guidelines for the Prevention, Detection and Management of Chronic Heart Failure in Australia, 2006.* Vol 194.; 2011. http://eutils.ncbi.nlm.nih.gov/entrez/eutils/elink.fcgi?dbfrom=pubmed&id=21495941&retmode=ref&cmd=prlinks.
4. Forst T, Hanefeld M, Jacob S, et al. Association of sulphonylurea treatment with all-cause and cardiovascular mortality: a systematic review and meta-analysis of observational studies. *Diab Vasc Dis Res*. 2013;10(4):302-314. doi:10.1177/1479164112465442.
5. Eurich DT, McAlister FA, Blackburn DF, et al. Benefits and harms of antidiabetic agents in patients with diabetes and heart failure: systematic review. *BMJ*. 2007;335(7618):497. doi:10.1136/bmj.39314.620174.80.
6. Smooke S, Horwich TB, Fonarow GC. Insulin-treated diabetes is associated with a marked increase in mortality in patients with advanced heart failure. *Am Heart J*. 2005;149(1):168-174. doi:10.1016/j.ahj.2004.07.005.
7. Scirica BM, Bhatt DL, Braunwald E, et al. Saxagliptin and cardiovascular outcomes in patients with type 2 diabetes mellitus. *N Engl J Med*. 2013;369(14):1317-1326. doi:10.1056/NEJMoa1307684.
8. Ryden L, Grant PJ, Anker SD, et al. ESC guidelines on diabetes, pre-diabetes, and cardiovascular diseases developed in collaboration with the EASD. *Eur Heart J*. 2013;34(39):3035-3087. doi:10.1093/eurheartj/eht108.
9. Zinman B, Wanner C, Lachin JM, et al. Empagliflozin, Cardiovascular Outcomes, and Mortality in Type 2 Diabetes. *N Engl J Med*. 2015;373(22):2117-2128. doi:10.1056/NEJMoa1504720.

For peer review only

10. Fitchett D, Zinman B, Wanner C, et al. Heart failure outcomes with empagliflozin in patients with type 2 diabetes at high cardiovascular risk: results of the EMPA-REG OUTCOME(R) trial. *Eur Heart J*. 2016;37(19):1526-1534. doi:10.1093/eurheartj/ehv728.

11. McMurray J. EMPA-REG - The "diuretic hypothesis." *J Diabetes Complications*. 2016;30(1):3-4. doi:10.1016/j.jdiacomp.2015.10.012.

12. Heerspink HJL, Perkins BA, Fitchett DH, Husain M, Cherney DZI. Sodium Glucose Cotransporter 2 Inhibitors in the Treatment of Diabetes Mellitus: Cardiovascular and Kidney Effects, Potential Mechanisms, and Clinical Applications. *Circulation*. 2016;134(10):752-772. doi:10.1161/CIRCULATIONAHA.116.021887.

13. Skrtic M, Cherney DZI. Sodium-glucose cotransporter-2 inhibition and the potential for renal protection in diabetic nephropathy. *Curr Opin Nephrol Hypertens*. 2015;24(1):96-103 8p. doi:10.1097/MNH.0000000000000084.

14. Cherney DZI, Perkins BA, Soleymanlou N, et al. Renal hemodynamic effect of sodium-glucose cotransporter 2 inhibition in patients with type 1 diabetes mellitus. *Circulation*. 2014;129(5):587-597. doi:10.1161/CIRCULATIONAHA.113.005081.

15. Thomas MC. Renal effects of dapagliflozin in patients with type 2 diabetes. *Ther Adv Endocrinol Metab*. 2014;5(3):53-61. doi:10.1177/2042018814544153.

16. Sha S Heise T, Natarajan J, Farrell K, Wang SS, Sica D, Rothenberg P, Plum-Morschel L PD. Effect of the sodium glucose co-transporter 2 inhibitor canagliflozin on plasma volume in patients with type 2 diabetes mellitus. *Diabetes Obes Metab*. 2014;16(11):1087. http://ovidsp.ovid.com/ovidweb.cgi?T=JS&PAGE=reference&D=cctr&NEWS=N&AN=CN-01115818.

17. Heise T, Macha S, Hantel S, Pinnetti S, Seman L, Woerle HJ. Safety, tolerability, pharmacokinetics and pharmacodynamics following 4 weeks' treatment with empagliflozin once daily in patients with type 2 diabetes. *Diabetes, Obes Metab*. 2013;15:613-621. doi:10.1111/dom.12073.

18. Sairaku A, Nakano Y, Kihara Y. Increased urine output by ipragliflozin in a non-diabetic patient with a diuretic-resistant heart failure. *Int J Cardiol*. 2015;180:42-43. doi:10.1016/j.ijcard.2014.11.138.

19. Wanner C, Inzucchi SE, Lachin JM, et al. Empagliflozin and Progression of Kidney Disease in Type 2 Diabetes. *N Engl J Med*. 2016;375(4):323-334. doi:10.1056/NEJMoa1515920.

20. Veldhuisen DJ Van, Ruilope LM, Maisel AS, Damman K. Clinical update Biomarkers of renal injury and function : diagnostic , prognostic and therapeutic implications in heart failure. 2016:2577-2585. doi:10.1093/eurheartj/ehv588.

21. Lang C, Choy A, Rahman A, Struthers A. Renal effects of low dose prazosin in patients with congestive heart failure. *Eur Heart J*. 1993;14(9):1245-1252.

For peer review only - http://bmjopen.bmj.com/site/about/guidelines.xhtml

http://onlinelibrary.wiley.com/o/cochrane/clcentral/articles/819/CN-00096819/frame.html.

22. Goldsmith S, Gilbertson D, Mackedanz S, Swan S. Renal effects of conivaptan, furosemide, and the combination in patients with chronic heart failure. *J Card Fail*. 2011;17(12):982-989. doi:10.1016/j.cardfail.2011.08.012.

23. List JF, Woo V, Morales E, Tang W, Fiedorek FT. Sodium-glucose cotransport inhibition with dapagliflozin in type 2 diabetes. *Diabetes Care*. 2009;32(4):650-657. doi:10.2337/dc08-1863.

24. Mudaliar S, Alloju S, Henry RR. Can a shift in fuel energetics explain the beneficial cardiorenal outcomes in the EMPA-REG OUTCOME study? a unifying hypothesis. *Diabetes Care*. 2016;39(7):1115-1122. doi:10.2337/dc16-0542.

25. Bebawy JF, Ramaiah VK, Zeeni C, Hemmer LB, Koht A, Gupta DK. The effect of furosemide on intravascular volume status and electrolytes in patients receiving mannitol: an intraoperative safety analysis. *J Neurosurg Anesthesiol*. 2013;25(1):51-54. doi:10.1097/ANA.0b013e318269c335.

26. Sirivella S, Gielchinsky I, Parsonnet V. Mannitol, furosemide, and dopamine infusion in postoperative renal failure complicating cardiac surgery. *Ann Thorac Surg*. 2000;69(2):501-506. doi:10.1016/S0003-4975(99)01298-9.

27. Turagam MK, Velagapudi P, Kalra AS, Ramalingam VS, Sinnakirouchenan R, Holley JL. Outcomes of furosemide-mannitol infusion in hospitalized patients with heart failure: an observational single-center cohort study of 122 patients. *Int J Cardiol*. 2011;151(2):232-234. doi:10.1016/j.ijcard.2011.06.044.

For peer review only

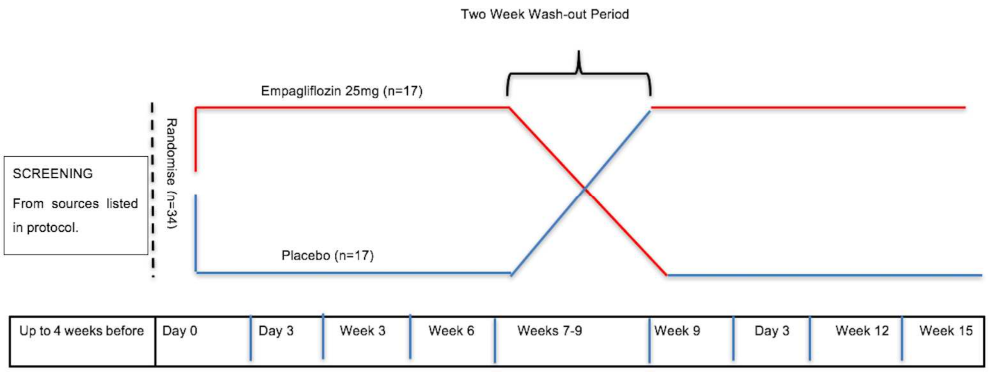

Figure 1: RECEDE-CHF Trial Design

peer review only

e.g. Time	Interval	Urine		Blood	
0730	- 30				Arrives fasted. 2 x cannula sited
0745	- 15				15mls/kg oral water load over 15 minutes
0800	0				
0830	+ 30	Void urine. Water intake equal to urine volume.			
0900	+ 60	Void urine. Water intake equal to urine volume			
0930	+ 90	Void urine. Water intake equal to urine volume.			
0945	+ 105		B1 (90-120 mins)	Bloods (serum B1) for U&Es & osmolality	
1000	+ 120	Void urine. Water intake equal to urine volume. Collect urine (urine B1) for volume, sodium, creatinine & osmolality.			
1015	+ 135		B2 (120-150 mins)	Bloods (serum B2) for U&Es & osmolality	
1030	+ 150	Void urine. Water intake equal to urine volume. Collect urine (urine B2) for volume, sodium, creatinine & osmolality			IMP administered: Oral tablet (empagliflozin 20mg or placebo)
1045	+ 165		D1 (150-180 mins)	Bloods (serum D1) for U&Es & osmolality	
1100	+ 180	Void urine. Water intake equal to urine volume. Collect urine for volume, sodium, creatinine & osmolality			
1115	+ 195		D2 (180-210 mins)	Bloods (serum D2) for U&Es & osmolality	
1130	+ 210	Void urine. Water intake equal to urine volume. Collect urine for volume, sodium, creatinine & osmolality			IV furosemide administered: half participant's screening dose
1145	+ 225		F1 (210 – 240 mins)	Bloods (serum F1) for U&Es & osmolality	
1200	+ 240	Void urine. Water intake equal to urine volume. Collect urine for volume, sodium, creatinine & osmolality			
1215	+ 255		F2 (240-270 mins)	Bloods (serum F2) for U&Es & osmolality	
1230	+ 270	Collect urine for volume, sodium, creatinine & osmolality.			**END OF STUDY PERIOD**

Figure 2: Protocol for Renal Physiology Test Days

only

For peer review only - http://bmjopen.bmj.com/site/about/guidelines.xhtml

SPIRIT

STANDARD PROTOCOL ITEMS: RECOMMENDATIONS FOR INTERVENTIONAL TRIALS

SPIRIT 2013 Checklist: Recommended items to address in a clinical trial protocol and related documents*

Section/item	Item No	Description	Addressed on page number
**Administrative information**			
Title	1	Descriptive title identifying the study design, population, interventions, and, if applicable, trial acronym	1
Trial registration	2a	Trial identifier and registry name. If not yet registered, name of intended registry	2
	2b	All items from the World Health Organization Trial Registration Data Set	
Protocol version	3	Date and version identifier	1
Funding	4	Sources and types of financial, material, and other support	21
Roles and responsibilities	5a	Names, affiliations, and roles of protocol contributors	21
	5b	Name and contact information for the trial sponsor	1
	5c	Role of study sponsor and funders, if any, in study design; collection, management, analysis, and interpretation of data; writing of the report; and the decision to submit the report for publication, including whether they will have ultimate authority over any of these activities	21
	5d	Composition, roles, and responsibilities of the coordinating centre, steering committee, endpoint adjudication committee, data management team, and other individuals or groups overseeing the trial, if applicable (see Item 21a for data monitoring committee)	

For peer review only - http://bmjopen.bmj.com/site/about/guidelines.xhtml

Introduction

Background and rationale	6a	Description of research question and justification for undertaking the trial, including summary of relevant studies (published and unpublished) examining benefits and harms for each intervention	____4-6_______
	6b	Explanation for choice of comparators	_____4-5_______
Objectives	7	Specific objectives or hypotheses	___13_______
Trial design	8	Description of trial design including type of trial (eg, parallel group, crossover, factorial, single group), allocation ratio, and framework (eg, superiority, equivalence, noninferiority, exploratory)	______6-7______

Methods: Participants, interventions, and outcomes

Study setting	9	Description of study settings (eg, community clinic, academic hospital) and list of countries where data will be collected. Reference to where list of study sites can be obtained	______2_______
Eligibility criteria	10	Inclusion and exclusion criteria for participants. If applicable, eligibility criteria for study centres and individuals who will perform the interventions (eg, surgeons, psychotherapists)	_____11-12_____
Interventions	11a	Interventions for each group with sufficient detail to allow replication, including how and when they will be administered	___2, 13-14_____
	11b	Criteria for discontinuing or modifying allocated interventions for a given trial participant (eg, drug dose change in response to harms, participant request, or improving/worsening disease)	_____14________
	11c	Strategies to improve adherence to intervention protocols, and any procedures for monitoring adherence (eg, drug tablet return, laboratory tests)	______8_______
	11d	Relevant concomitant care and interventions that are permitted or prohibited during the trial	______12_______
Outcomes	12	Primary, secondary, and other outcomes, including the specific measurement variable (eg, systolic blood pressure), analysis metric (eg, change from baseline, final value, time to event), method of aggregation (eg, median, proportion), and time point for each outcome. Explanation of the clinical relevance of chosen efficacy and harm outcomes is strongly recommended	____2, 13_______

For peer review only - http://bmjopen.bmj.com/site/about/guidelines.xhtml

Participant timeline	13	Time schedule of enrolment, interventions (including any run-ins and washouts), assessments, and visits for participants. A schematic diagram is highly recommended (see Figure)	____6-7, 8-9_____
Sample size	14	Estimated number of participants needed to achieve study objectives and how it was determined, including clinical and statistical assumptions supporting any sample size calculations	_____14________
Recruitment	15	Strategies for achieving adequate participant enrolment to reach target sample size	______14_______
**Methods: Assignment of interventions (for controlled trials)**			
Allocation:			
Sequence generation	16a	Method of generating the allocation sequence (eg, computer-generated random numbers), and list of any factors for stratification. To reduce predictability of a random sequence, details of any planned restriction (eg, blocking) should be provided in a separate document that is unavailable to those who enrol participants or assign interventions	______10_______
Allocation concealment mechanism	16b	Mechanism of implementing the allocation sequence (eg, central telephone; sequentially numbered, opaque, sealed envelopes), describing any steps to conceal the sequence until interventions are assigned	_____12________
Implementation	16c	Who will generate the allocation sequence, who will enrol participants, and who will assign participants to interventions	___11-13_______
Blinding (masking)	17a	Who will be blinded after assignment to interventions (eg, trial participants, care providers, outcome assessors, data analysts), and how	______12_______
	17b	If blinded, circumstances under which unblinding is permissible, and procedure for revealing a participant's allocated intervention during the trial	______12_______
**Methods: Data collection, management, and analysis**			
Data collection methods	18a	Plans for assessment and collection of outcome, baseline, and other trial data, including any related processes to promote data quality (eg, duplicate measurements, training of assessors) and a description of study instruments (eg, questionnaires, laboratory tests) along with their reliability and validity, if known. Reference to where data collection forms can be found, if not in the protocol	_____15________

For peer review only - http://bmjopen.bmj.com/site/about/guidelines.xhtml

	18b	Plans to promote participant retention and complete follow-up, including list of any outcome data to be collected for participants who discontinue or deviate from intervention protocols	______________
Data management	19	Plans for data entry, coding, security, and storage, including any related processes to promote data quality (eg, double data entry; range checks for data values). Reference to where details of data management procedures can be found, if not in the protocol	_____15________
Statistical methods	20a	Statistical methods for analysing primary and secondary outcomes. Reference to where other details of the statistical analysis plan can be found, if not in the protocol	_____15______
	20b	Methods for any additional analyses (eg, subgroup and adjusted analyses)	______________
	20c	Definition of analysis population relating to protocol non-adherence (eg, as randomised analysis), and any statistical methods to handle missing data (eg, multiple imputation)	______________
**Methods: Monitoring**			
Data monitoring	21a	Composition of data monitoring committee (DMC); summary of its role and reporting structure; statement of whether it is independent from the sponsor and competing interests; and reference to where further details about its charter can be found, if not in the protocol. Alternatively, an explanation of why a DMC is not needed	____16________
	21b	Description of any interim analyses and stopping guidelines, including who will have access to these interim results and make the final decision to terminate the trial	______________
Harms	22	Plans for collecting, assessing, reporting, and managing solicited and spontaneously reported adverse events and other unintended effects of trial interventions or trial conduct	____16-17_____
Auditing	23	Frequency and procedures for auditing trial conduct, if any, and whether the process will be independent from investigators and the sponsor	______16_______
**Ethics and dissemination**			
Research ethics approval	24	Plans for seeking research ethics committee/institutional review board (REC/IRB) approval	_____17_____

For peer review only - http://bmjopen.bmj.com/site/about/guidelines.xhtml

Protocol amendments	25	Plans for communicating important protocol modifications (eg, changes to eligibility criteria, outcomes, analyses) to relevant parties (eg, investigators, REC/IRBs, trial participants, trial registries, journals, regulators)	______17________
Consent or assent	26a	Who will obtain informed consent or assent from potential trial participants or authorised surrogates, and how (see Item 32)	_________10_____
	26b	Additional consent provisions for collection and use of participant data and biological specimens in ancillary studies, if applicable	______________
Confidentiality	27	How personal information about potential and enrolled participants will be collected, shared, and maintained in order to protect confidentiality before, during, and after the trial	_____17________
Declaration of interests	28	Financial and other competing interests for principal investigators for the overall trial and each study site	_____21________
Access to data	29	Statement of who will have access to the final trial dataset, and disclosure of contractual agreements that limit such access for investigators	______18_______
Ancillary and post-trial care	30	Provisions, if any, for ancillary and post-trial care, and for compensation to those who suffer harm from trial participation	______________
Dissemination policy	31a	Plans for investigators and sponsor to communicate trial results to participants, healthcare professionals, the public, and other relevant groups (eg, via publication, reporting in results databases, or other data sharing arrangements), including any publication restrictions	________17______
	31b	Authorship eligibility guidelines and any intended use of professional writers	______________
	31c	Plans, if any, for granting public access to the full protocol, participant-level dataset, and statistical code	______________
**Appendices**			
Informed consent materials	32	Model consent form and other related documentation given to participants and authorised surrogates	__not included___
Biological specimens	33	Plans for collection, laboratory evaluation, and storage of biological specimens for genetic or molecular analysis in the current trial and for future use in ancillary studies, if applicable	______________

For peer review only - http://bmjopen.bmj.com/site/about/guidelines.xhtml

*It is strongly recommended that this checklist be read in conjunction with the SPIRIT 2013 Explanation & Elaboration for important clarification on the items. Amendments to the protocol should be tracked and dated. The SPIRIT checklist is copyrighted by the SPIRIT Group under the Creative Commons "Attribution-NonCommercial-NoDerivs 3.0 Unported" license.

For peer review only

For peer review only - http://bmjopen.bmj.com/site/about/guidelines.xhtml
