# Supplementary material for: Renal and Cardiovascular Effects of sodium–glucose cotransporter 2 (SGLT2) inhibition in combination with loop Diuretics in diabetic patients with Chronic Heart Failure (RECEDE-CHF): protocol for a randomised controlled double-blind cross-over trial
Source: BMJ Open. 2017 Oct 16;7(10):e018097. doi: 10.1136/bmjopen-2017-018097 (PMC5652490; doi:10.1136/bmjopen-2017-018097)
Supplement: Reviewer comments [file bmjopen-2017-018097.reviewer_comments.pdf]

PEER REVIEW HISTORY

BMJ Open publishes all reviews undertaken for accepted manuscripts. Reviewers are asked to complete a checklist review form (**http://bmjopen.bmj.com/site/about/resources/checklist.pdf**) and are provided with free text boxes to elaborate on their assessment. These free text comments are reproduced below.

ARTICLE DETAILS

**TITLE (PROVISIONAL)**	The Renal and Cardiovascular Effect of Sodium-Glucose co-Transporter 2 (SGLT2) inhibition in combination with Loop Diuretics in Diabetic patients with Chronic Heart Failure (RECEDE-CHF): Protocol for a Randomised Controlled Double-Blind Crossover Trial.
**AUTHORS**	Mordi, Natalie; Mordi, Ify; Singh, Jagdeep; Baig, Fatima; Choy, Anna-Maria; McCrimmon, Rory; Struthers, Allan; Lang, Chim C.

This article was not externally reviewed at BMJ Open. Protocols that have been independently assessed prior to submission to BMJ Open are usually fast-tracked to publication on the grounds that further substantial changes will not be possible. This independent assessment will usually be external, independent review for both a substantial grant award from a non-commercial or government funder and ethics approval.
